# Evidence of Superior and Inferior Sinoatrial Nodes in the Mammalian Heart

**DOI:** 10.1016/j.jacep.2020.09.012

**Published:** 2020-11-25

**Authors:** Jaclyn A. Brennan, Qing Chen, Anna Gams, Jhansi Dyavanapalli, David Mendelowitz, Weiqun Peng, Igor R. Efimov

**Affiliations:** a Department of Biomedical Engineering, George Washington University School of Engineering and Applied Sciences, Washington, DC; b Department of Physics, George Washington University Columbian College of Art and Sciences, Washington, DC; c Department of Pharmacology and Physiology, George Washington University School of Medicine and Health Sciences, Washington, DC.

**Keywords:** arrhythmias, sinoatrial node, optical mapping, pacemaker

## Abstract

**OBJECTIVES:**

This study sought to investigate the shift of leading pacemaker locations in healthy and failing mammalian hearts over the entire range of physiological heart rates (HRs), and to molecularly characterize spatial regions of spontaneous activity.

**BACKGROUND:**

A normal heartbeat originates as an action potential in a group of pacemaker cells known as the sinoatrial node (SAN), located near the superior vena cava. HRs and the anatomical site of origin of pacemaker activity in the adult heart are known to dynamically change in response to various physiological inputs, yet the mechanism of this pacemaker shift is not well understood.

**METHODS:**

Optical mapping was applied to ex vivo rat and human isolated right atrial tissues, and HRs were modulated with acetylcholine and isoproterenol. RNA sequencing was performed on tissue areas that elicited spontaneous activity, and comparisons were made to neighboring myocardial tissues.

**RESULTS:**

Functional and molecular evidence identified and confirmed the presence of 2 competing right atrial pacemakers localized near the superior vena cava and the inferior vena cava—the superior SAN (sSAN) and inferior SAN (iSAN), respectively—which preferentially control the fast and slow HRs. Both of these regions were evident in non-failing rat and human hearts and maintained spontaneous activity in the rat heart when physically separated from one another. Molecular analysis of these 2 pacemaker regions revealed unique but similar transcriptional profiles, suggesting iSAN dominance when the sSAN is silent.

**CONCLUSIONS:**

The presence of 2 spatially distinct dominant pacemakers, sSAN and iSAN, in the mammalian heart clarifies previous identification of migrating pacemakers and corresponding changes in P-wave morphology in mammalian species.

For years, the “textbook” location of the sinoatrial node (SAN) has generally been depicted at the junction of the superior vena cava (SVC) and the right atrium (RA) (Figure 1A). However, the site of origin of the electrical activity of the heart— also referred to as the leading pacemaker site—has been shown to anatomically shift with various types of interventions that modify heart rate (HR) (1,2). Over the past century, spatial shifts of the dominant leading pacemaker site have been identified in the mouse (3), rabbit (4–6), dog (7), and human (8) heart, with influence from external factors such as sympathetic and parasympathetic stimulation, cardiac glycosides, temperature changes, and alterations of extracellular Na^+^, K^+^, Ca^2+^, and Cl^–^ concentrations (2,9). Although pacemaker cells are known to be widely distributed throughout the entire venae cavae region between the crista terminalis and interatrial septum (10), the rationale for the observed anatomical shifts of spontaneous electrical activity are still incompletely understood, and the demarcation of the center and periphery (or head and tail) of the SAN continues to be ill-defined.

Optical mapping has evolved over the years as a valuable technique for investigating cardiac electrophysiology (11). Utilizing voltage-sensitive dyes that bind to cell membranes, it offers high spatial and temporal resolutions for isolated preparations of complex electrically active tissues (12). Traditional electrophysiological methods for investigating the SAN involve string galvanometers, intracellular microelectrodes, extracellular bipolar or multielectrode arrays, and unipolar surface electrograms. However, for intricate cardiac tissues such as the SAN, optical mapping is arguably one of the best technologies currently available for identifying beat-to-beat changes over the full range of physiological conditions, a concept that we are only just beginning to explore.

Given the large but currently conflicting published data on the anatomical shift of the leading pacemaker site, it was the aim of this study to quantify changes in SAN dynamics using optical mapping techniques on a beat-to-beat basis during changes in HR. HRs are known to dynamically change in vivo to adjust cardiac output to meet physiological demands, as they are altered by factors such as autonomic tone, exercise, stress, temperature, and circadian variability (2). HRs can also change with age, dysfunction of the SAN, and various illnesses such as myocardial infarctions and heart failure (13,14). Therefore, we sought to: 1) elucidate temporal and spatial changes of the leading pacemaker in ex vivo healthy and failing SAN preparations over the range of physiological HRs using optical mapping techniques; and 2) characterize the specific gene expression profile of the notable pacemaking regions in comparison with neighboring atrial tissues. Rat and human hearts were chosen for this study because their SANs have been relatively less studied in the field to date (15).

## METHODS

### ETHICAL APPROVAL.

All animal procedures were completed in agreement with the George Washington University’s institutional guidelines and in compliance with suggestions from the panel of Euthanasia of the American Veterinary Medical Association and the National Institutes of Health Guide for the Care and Use of Laboratory Animals. Procurement of de-identified donor human hearts rejected for transplant were approved for research by the Institutional Review Board (Office of Human Research) at the George Washington University (Washington, DC) and by the organ procurement organization Washington Regional Transplant Community (Falls Church, Virginia).

### RAT MODEL OF HEART FAILURE.

Ascending aortic constriction was performed on 1-week-old male Sprague-Dawley rat pups to induce left ventricular hypertrophy. Briefly, the aorta of deeply anesthetized neonatal rats was constricted with a silk suture tied around a custom-made 20-gauge spacer. After ligation, the spacer was removed and the chest of the animal sutured shut. Rats were monitored daily until animals were sacrificed for experiments.

### RAT HEART EXCISION AND ISOLATION OF THE RIGHT ATRIA.

For functional studies, healthy (n = 8) and failing (n = 6) adult male rats (8 to 12 weeks of age) were deeply anesthetized until unconscious. Hearts were quickly excised, cannulated, and placed in a constant-pressure Langendorff system with warmed (37°C) and oxygenated Tyrode’s solution. The RA of each heart was isolated, opened, and pinned to a Sylgard-coated chamber for visualization of the SAN and superfused with warmed Tyrode’s solution to maintain viability.

### HUMAN HEART PROCUREMENT AND ISOLATION OF THE RA.

De-identified human hearts were procured from the Washington Regional Transplant Community and arrested in an ice-cold cardioplegic solution upon excision from the donor (Supplemental Table 1). Hearts were then shipped on ice to our laboratory, where the entire RA was carefully isolated, and the right coronary artery was cannulated with a custom flexible plastic cannula for optical mapping (n = 3). All major transected arteries were tied off, and the tissue was stretched across a custom frame and transferred to a vertical bath of warmed and oxygenated Tyrode’s solution for dual-sided optical mapping of both the epicardial and endocardial surfaces. Adequate perfusion (60 to 80 mm Hg) was maintained for the duration of the experiment.

### OPTICAL MAPPING.

Both the rat and human isolated RA preparations were electromechanically uncoupled with blebbistatin (5 to 10 μM) and fluorescently stained with di-4-ANEPPS. Optical action potentials (OAPs) were captured with high-resolution cameras (100 × 100 pixels, 1- to 2-kHz sampling frequency, Ultima-L or MiCAM05 CMOS cameras, SciMedia, Costa Mesa, California). For each recording, tissues were illuminated with a 520-nm light-emitting diode (Prizmatix, Holon, Israel), and emitted fluorescence was captured through a 650-nm long-pass filter (Thorlabs, Newton, New Jersey). Rat tissues were treated with increasing dosages of acetylcholine (ACh) (1, 10, 100, 500, and 1,000 μM; Millipore-Sigma, Burlington, Massachusetts) and increasing dosages of isoproterenol (1, 10, 100, 500, and 1,000 nM; Tocris Bioscience, Ellisville, Missouri), with a washout step in-between the 2 drugs. Human tissues were treated with 500-nM ACh and 100-nM ISO, also with a washout step in-between. Optical recordings were taken during normal sinus rhythm and under the influence of each dosage of drug (usually 5 to 10 min after administration of each dose), and HRs were monitored with sensing electrodes for pseudo-electrocardiography recodings with LabChart (AD Instruments, Sydney, Australia).

### IDENTIFICATION OF THE LEADING PACEMAKER LOCATION.

**FIGURE 1:**
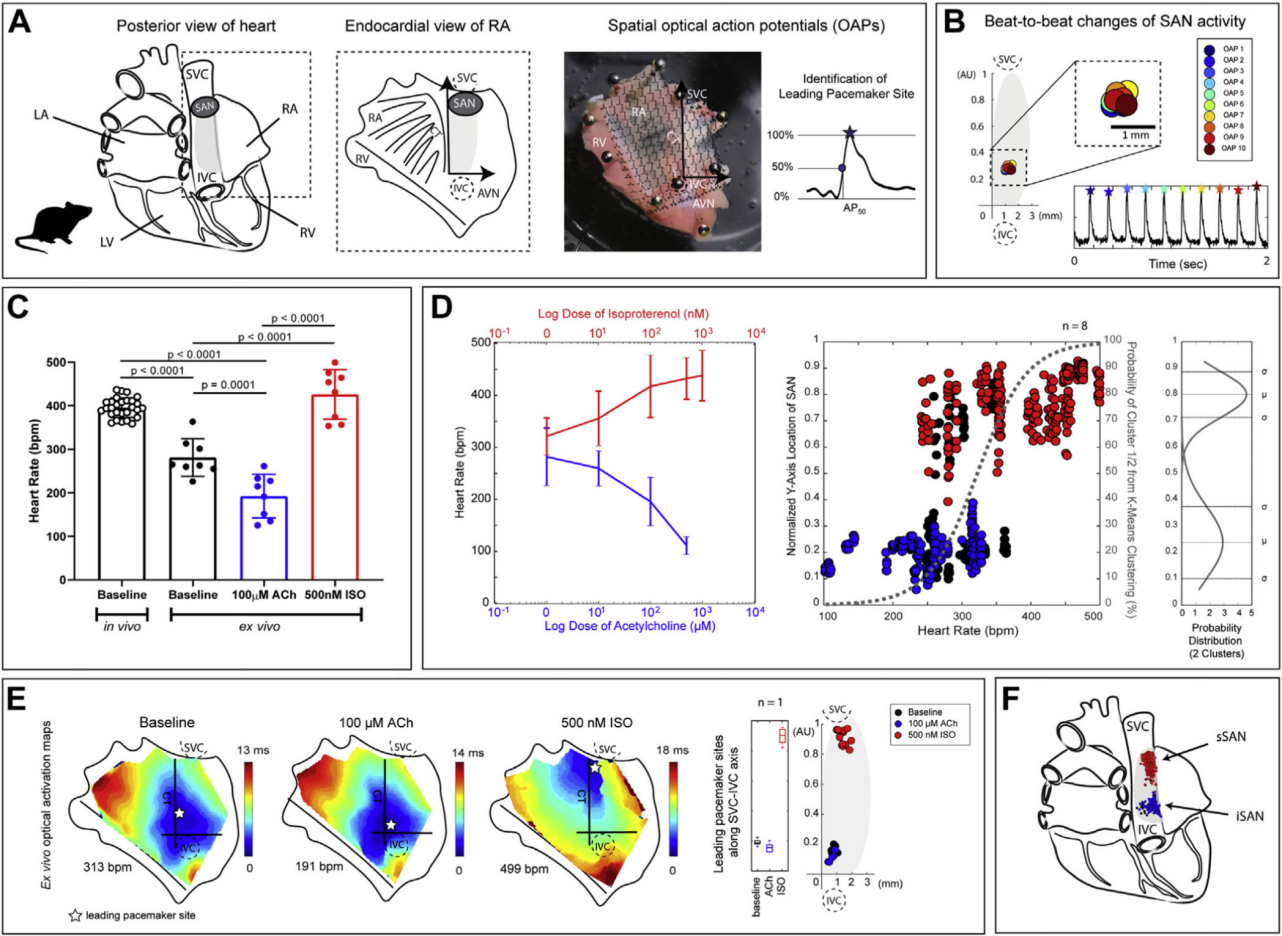
Functional Evidence of sSAN and iSAN in the Isolated Ex Vivo Rat Heart **(A) (Left)** Schematic of the posterior view of the rat heart with the “textbook” location of the sinoatrial node (SAN) located at the junction of the superior vena cava (SVC) and right atria (RA). **(Middle)** Schematic of the endocardial view of the isolated RA. **(Right)** Representative image of an isolated RA preparation of the rat heart overlaid with optical action potentials (OAPs) (time 100 ms). The leading pacemaker site is identified as the location within the preparation that displays the earliest activation time as identified during the upstroke by 50% of the OAP amplitude (AP_50_). **(B)** Representative OAPs for 10 consecutive beats over a 2-s optical recording. Points are replotted onto a new y-axis in arbitrary units (AU) along the crista terminalis (CT) between the inferior vena cava (IVC) to the SVC and normalized between 0 and 1. **(C)** Intrinsic heart rates (HRs) of in vivo (8 to 12 weeks of age; n = 7) rat hearts and ex vivo (8 to 12 weeks of age; n = 8) rat SANs. **(D) (Left)** HR changes caused by the administration of ACh or ISO in all rats (n = 8). **(Middle)** Normalized locations of leading pacemaker sites under baseline conditions **(black)**, ACh **(blue)**, and ISO **(red)** plotted against corresponding HRs. Points were assigned to 1 of 2 cluster locations (superior or inferior) and fitted with a logistic regression curve using k-means clustering analysis **(gray dashed line)**. **(Right)** The two clusters are fitted with a normal probability distribution (μ = mean, σ = standard deviation). Representative activation maps of an isolated RA tissue preparation during baseline sinus rhythm, high parasympathetic stimulation (100-mM acetylcholine chloride [ACh]), and high sympathetic stimulation (500-nM isoproterenol [ISO]). Points are replotted onto the new axis on a beat-to-beat basis and color-coded according to corresponding condition (baseline = **black**, ACh = **blue**, ISO = **red**). **(F)** New schematic of the posterior view of the rat heart with 2 spatially unique pacemakers: the superior SAN (sSAN) and inferior SAN (iSAN). A AVN = atrioventricular node; LA = left atria; RV = right ventricle.

A modified version of RHYTHM, a custom-made MATLAB 2016b program (The Math-Works, Natick, Massachusetts) designed for optical mapping data analysis, was used for the creation of activation maps. OAPs were filtered in space (3 × 3 or 5 × 5 pixel neighborhood for rat and human, respectively) and in time (low-pass Butterworth filter at 150 to 200 Hz), and activation maps were created by calculating 50% of the maximum OAP amplitudes (Figure 1A). The location of the earliest activation time was identified from its spatial neighbors, and this site within the SAN was automatically plotted on a beat-to-beat basis. To compare beats across hearts, each leading pacemaker site was replotted onto a new y-axis in arbitrary units (AU) between the inferior vena cava (IVC) (y = 0) and SVC (y = 1) along the crista terminalis, and onto a perpendicular x-axis (mm) (Figure 1B).

### RNA SEQUENCING SAMPLE PREPARATION AND ANALYSIS.

RNA sequencing was performed on tissues of age-matched healthy male rat hearts (n = 4) and healthy human donor hearts (n = 8) that were not used for functional experiments (Supplemental Table 1). The preparation of whole-transcriptome libraries and next-generation sequencing were conducted using the Illumina HiSeq System with paired-end 150 reads (Novogene, Sacramento, California). Raw reads were filtered by removing reads containing adapters, reads containing N >0.1% (N represents base that could not be determined), and low-quality reads. Rat sample reads were aligned to the reference genome Rattus norvegicus release 98 by HISAT2, and expression values were calculated using HTSeq v0.6.1 (16). Human sample reads were aligned to the reference genome hg38 using STAR (v2.5) (17). featureCounts was used to count the read numbers mapped of each gene (18). Differential expression analysis was carried out using DESeq2 (19). Differentially expressed genes (DEGs) were identified with the significance criterion p_adj_ < 0.05 for loose analysis, and p_adj_ < 0.01, fold change (FC) > 3 for stringent analysis. ClusterProfiler (20) was used for Gene Ontology (GO) analysis.

### STATISTICAL ANALYSIS.

Statistical analysis was performed using Prism 7 (GraphPad Software, San Diego, California). Significant differences were labeled with individual p values. Two different statistical tests were used in this study: 1- and 2-way analyses of variance with a Sidak post-test for multiple comparisons. Statistical tests were chosen and used as necessary and are indicated in the text.

## RESULTS

### EFFECTS OF PHARMACOLOGICAL INTERVENTION ON THE HEALTHY RAT SAN.

In healthy, conscious rats, the average in vivo resting HR was 395.50 ± 22.20 beats/min, while the average ex vivo baseline HR was 280.90 ± 43.00 beats/min (p < 0.0001) (Figure 1C). Baseline ex vivo HRs significantly dropped by 31.5% to 192.30 ± 50.15 beats/min with the addition of 100-μM ACh and increased from baseline by 57.0% to 426.00 ± 57.12 beats/min with the addition of 500-nM ISO (p = 0.0001 and p < 0.0001, respectively; 1-way analysis of variance). In the complete dose-response protocol of ACh and ISO (5 doses each) of the isolated rat RA, we observed the full range of physiologically possible ex vivo HRs (mean 324.89 ± 92.32 beats/min; range 102.47 to 502.09 beats/min) as well as corresponding spatial shifts of the leading pacemaker site on a beat-to-beat basis (Figure 1D). Two distinct clusters of pacemaking activity were observed along the normalized y-axis between the IVC and SVC, with preferential activity dictated by HR and drug influence. Specifically, one cluster centered close to the IVC at an average of 0.24 ± 0.14 AU, while the other, higher cluster centered around 0.80 ± 0.09 AU on the y-axis. Under baseline conditions (i.e., without any pharmacological influence), only 3 of the 8 rat RA preparations displayed leading pacemaker site activity from the higher cluster, corresponding to the traditional “textbook” location near the orifice of the SVC; the other 5 preparations exhibited a lower clustering of leading pacemaker activity close to the orifice of the IVC (Supplemental Figure 1). However, pacemaking activity always originated close to the SVC in the presence of ISO (or when HRs exceeded 400.00 beats/min) and from the IVC region in the presence of ACh (or when HRs were <250.00 beats/min). Representative activation maps under baseline conditions and in the presence of high muscarinic and adrenergic pharmacological stimulation are shown in Figure 1E. Based on the 2 observed clusters of intrinsic pacemaking activity, a complete schematic indicating the physiological presence of both a superior SAN (sSAN) and inferior SAN (iSAN) pacemaker region in the rat heart is displayed in Figure 1F.

### PACEMAKING ACTIVITY OF THE PHYSICALLY DISSECTED RAT sSAN AND iSAN.

**FIGURE 2:**
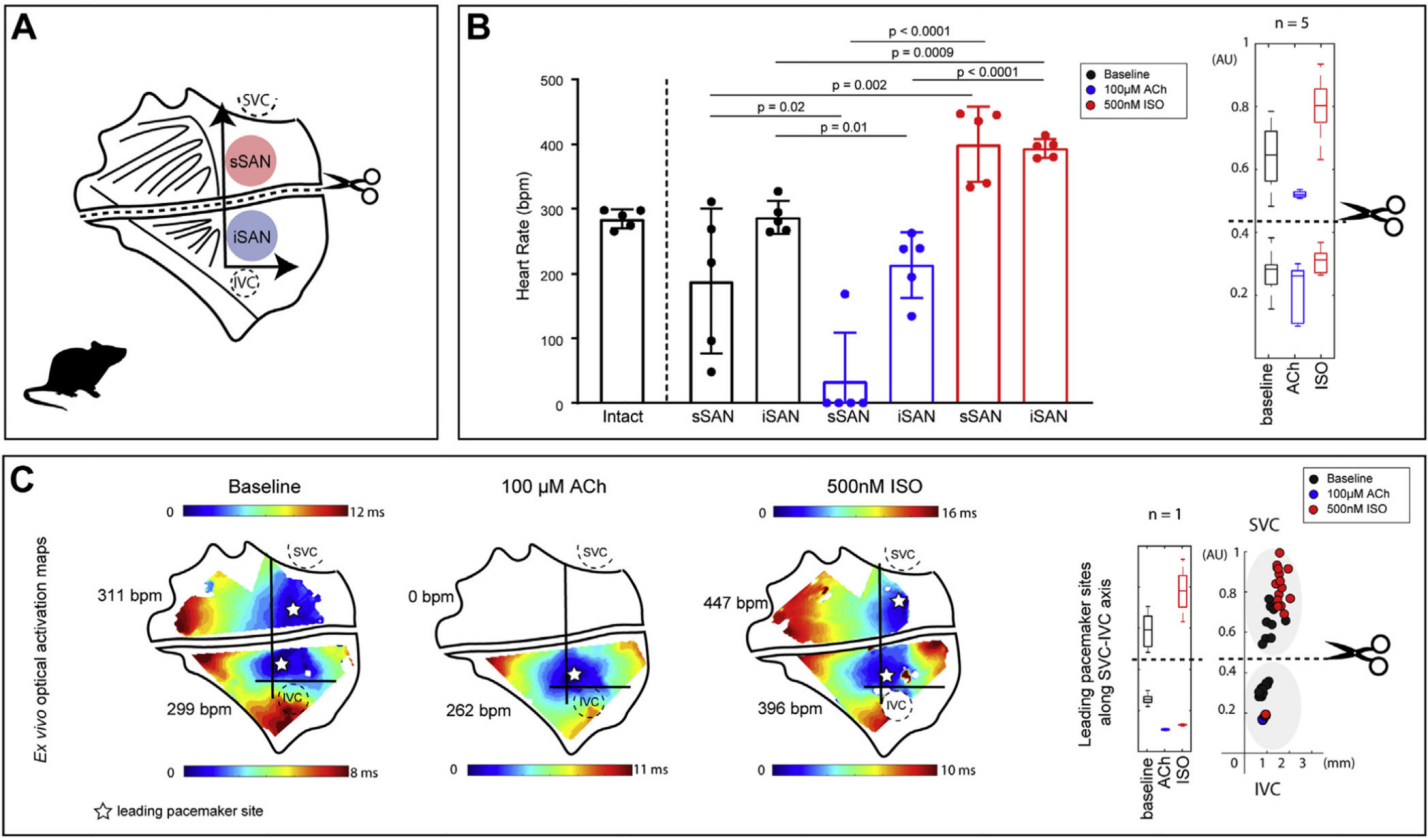
Functional Characterization of the Physically Separated Rat sSAN and iSAN **(A)** Schematic of the rat RA preparation surgically cut to isolate the sSAN and iSAN. **(B) (Left)** HR responses of ex vivo intact and physically separated SANs under baseline conditions **(black)** and after exposure to high parasympathetic stimulation (100-μM ACh **[blue]**) and high sympathetic stimulation (500 nM ISO **[red]**) (n = 5). **(Right)** Boxplots display the distribution of y-axis locations of leading pacemaker sites under each of the 3 experimental conditions after the physical separation of the SAN (n = 5). **(C) (Left)** Representative optical activation maps of a separated SAN during baseline sinus rhythm, high parasympathetic stimulation, and high sympathetic stimulation. **(Right)** Sites are replotted onto the new axis and color-coded according to corresponding condition (baseline = **black**, ACh = **blue**, ISO = **red**), with boxplots showing distribution of leading pacemaker locations (n = 1). Abbreviations as in Figure 1.

To examine the independent behavior of the newly identified sSAN and iSAN regions, we surgically separated the ex vivo rat SAN into 2 distinct tissues (Figure 2A). Though there were no significant differences between the HRs of the intact and separated tissues, the iSAN possessed intrinsic automaticity with a similar mean and small standard deviation as the intact preparation under baseline conditions (intact: 284.90 ± 14.46 beats/min; sSAN: 188.10 ± 112.30 beats/min; iSAN: 286.70 ± 25.50 beats/min; n = 5) (Figure 2B). With high parasympathetic stimulation (100-μM ACh), all but 1 cut sSAN became completely quiescent (Supplemental Figure 2), while the iSAN maintained an average HR of 213.60 ± 50.77 beats/min. Upon washout of ACh and administration of 500-nM ISO, all sSAN tissues regained automaticity and possessed intrinsic HRs comparable to the iSAN under ISO exposure (sSAN: 400.00 ± 58.20 beats/min; iSAN: 393.50 ± 14.63 beats/min; n = 5). Similar to the intact SAN, a hierarchical clustering distribution along the SVC-IVC axis is evident within each of the 2 separated tissues (i.e., pacemaker beats originate superiorly with sympathetic stimulation and inferiorly with parasympathetic stimulation). Representative optical activation maps from the surgically dissected SAN are shown in Figure 2C. In this particular preparation, the intact SAN exhibited a baseline HR of 290.00 beats/min prior to physical separation into 2 tissues.

### EFFECTS OF PHARMACOLOGICAL INTERVENTION ON THE FAILING RAT SAN.

**FIGURE 3:**
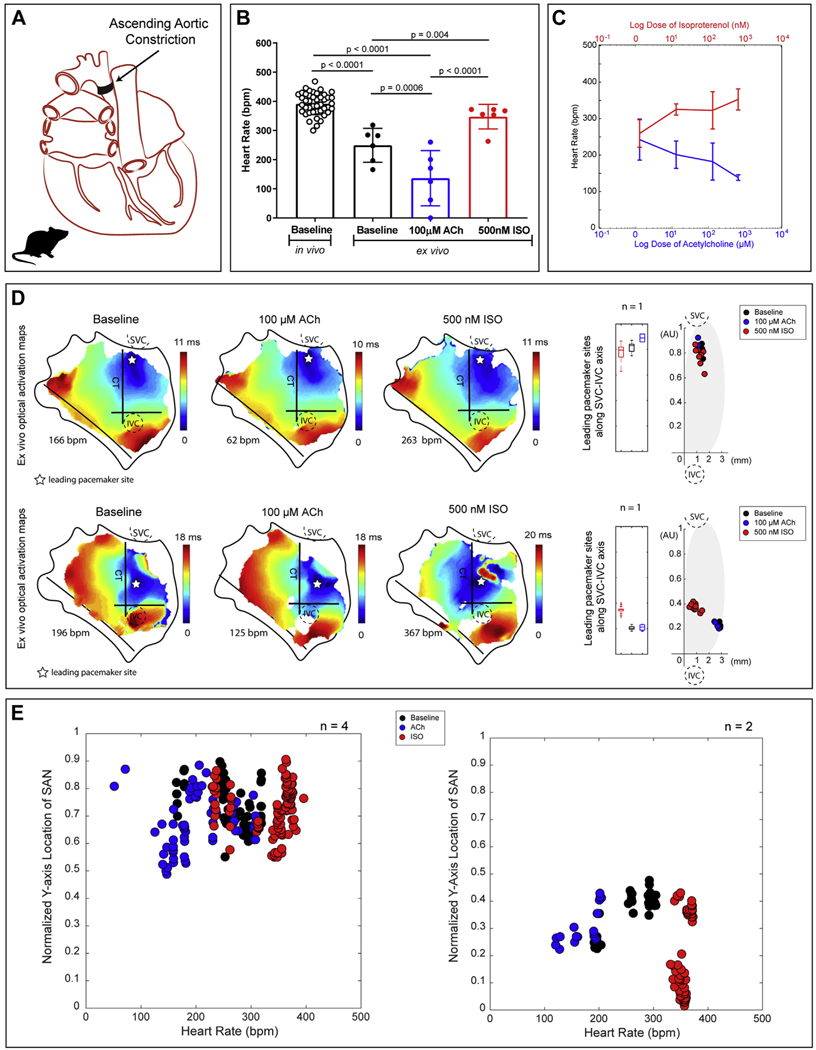
Functional Evidence of Only 1 Pacemaking Region, Either sSAN or iSAN, in the Failing Rat Heart **(A)** Schematic of the posterior view of a failing rat heart created by ascending aortic constriction. **(B)** Intrinsic HRs of in vivo (8 to 12 weeks of age; n = 11) failing rats and ex vivo (10 to 12 weeks of age; n = 6) end-stage failing rat SANs under baseline conditions (black), 100-µM ACh (blue), and 500-nM ISO (red). **(C)** HR changes with ACh and ISO in all ex vivo failing rat hearts (n = 6). **(D)** Representative activation maps of 2 isolated RA tissue preparation from heart failure rats during baseline sinus rhythm, high parasympathetic stimulation (100-µM ACh), and high sympathetic stimulation (500-nM ISO). Points are replotted onto the new axis on a beat-to-beat basis and color-coded according to corresponding condition (baseline = **black**, ACh = **blue**, ISO = **red**). **(E)** Four hearts displayed pacemaking activity from the sSAN alone during all treatment conditions, and 2 hearts displayed pacemaking activity from the iSAN alone during all treatment conditions. Abbreviations as in Figure 1.

In all end-stage failing rat hearts induced by ascending aortic constriction (Figure 3A), it was found that only 1 of the 2 pacemaker regions displays automaticity for each heart, despite ex vivo changes of HR by autonomic stimulation (mean 289.47 ± 71.40 beats/min; range 120.72 to 372.67 beats/min) (Figures 3B–E). As seen in Supplemental Figure 3 and Supplemental Table 2, clear significant anatomical differences in heart weights and inner and outer dimensions were observed between the normal and failing groups. However, between healthy and failing hearts, there were no significant differences between in vivo HRs (Supplemental Figure 3) or ex vivo sinus node recovery times (SNRTs) following a 12-pulse drive train at a pacing cycle length of 100 ms (Supplemental Figure 4). A prolonged SNRT is often coupled to SAN dysfunction, but this feature was not evident in the failing rats created for this study. Still, optical mapping data uniquely identified complete sSAN dominance in 66% of the failing hearts and complete iSAN dominance in 33% of the failing hearts (Figure 3E, Supplemental Figure 5).

### RNA SEQUENCING OF RAT sSAN AND iSAN.

**FIGURE 4:**
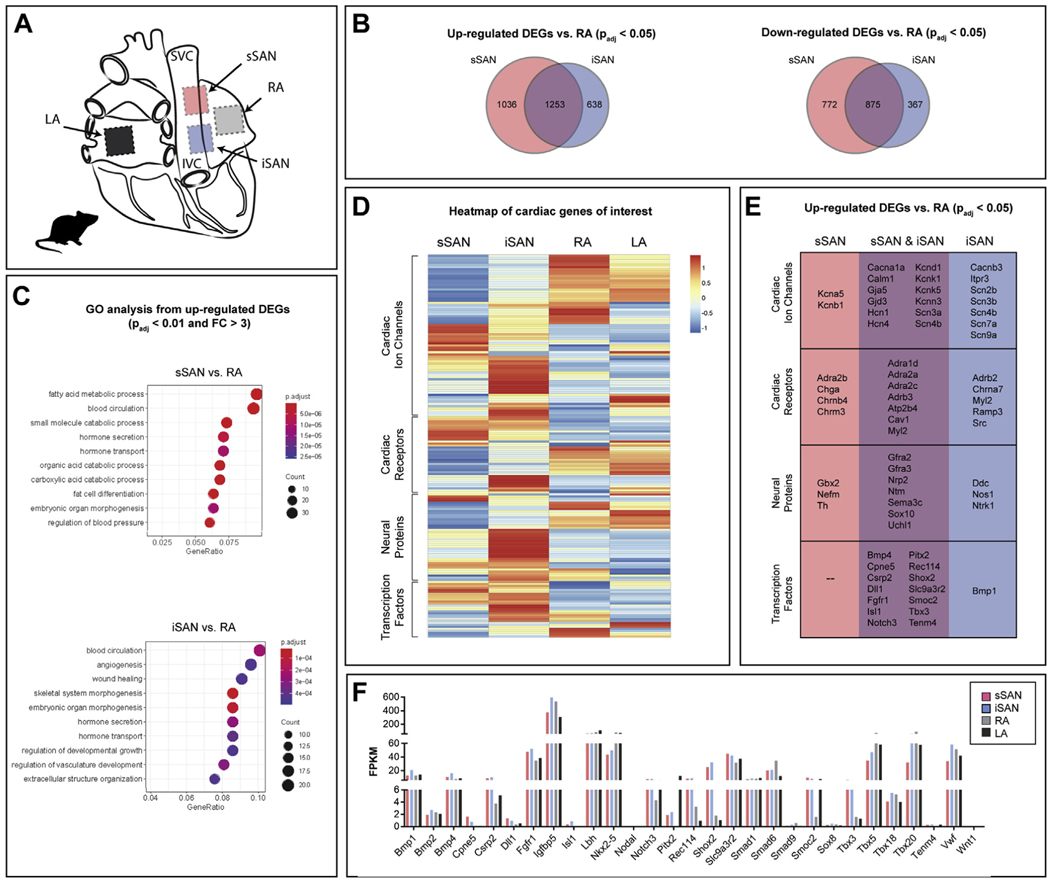
Molecular Characterization of the Rat sSAN and iSAN **(A)** Tissue segments of healthy rat hearts (n=4) were dissected for RNA sequencing from the sSAN, iSAN, RA, and LA. **(B)** Venn diagrams depict the number of up- and down-regulated differentially expressed genes (DEGs) (p_adj_ < 0.05) for the sSAN and iSAN as compared with the neighboring RA. **(C)** Most enriched Gene Ontology (GO) terms for up-regulated DEGs in the sSAN **(top)** and iSAN **(bottom)** as compared with the RA. **(D)** Heatmap showing the expression patterns of 4 categories of genes of interest for the different tissue regions. Genes within each category were subject to hierarchical clustering. Gene set enrichment analysis (GSEA) of the 4 gene sets found significant differences between sSAN and iSAN in the cardiac receptor group (data not shown). **(E)** List of up-regulated cardiac-specific DEGs for the sSAN and iSAN, as compared to the RA. **(F)** Fragments per kilobase of transcript per million mapped reads (FPKM) of cardiac-specific transcription factors across tissues. Abbreviations as in Figure 1.

RNA was isolated and sequenced from 4 regions of the healthy rat heart: sSAN, iSAN, RA, and left atrium (Figure 4A). Quantification of DEGs (p_adj_ < 0.05) identified that both the sSAN and iSAN contain more up-regulated than down-regulated DEGs when compared with the RA (Figure 4B). GO analyses of up-regulated DEGs spanning molecular functions, biological processes, and cellular components are displayed in Figure 4C. In addition to the unique GOs (sSAN: regulation of fatty acid metabolic processes, blood circulation, and small molecule catabolic processes; iSAN: regulation of blood circulation, angiogenesis, and wound healing), there is a higher overall prevalence of metabolic genes in the sSAN than the iSAN.

**FIGURE 5:**
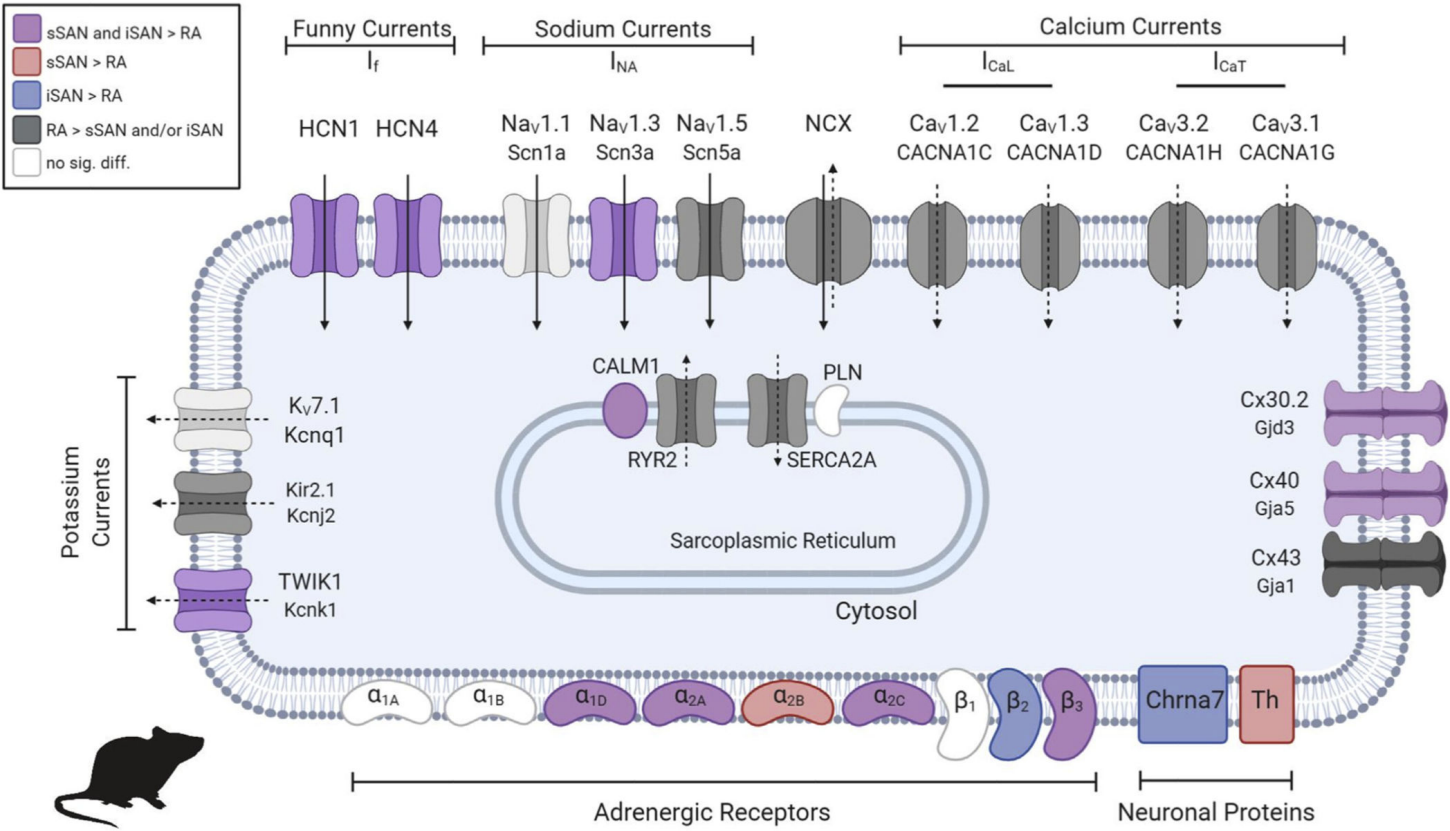
Ion Channels and Surface Receptors in the 2 Rat Pacemakers: sSAN and iSAN Channels which allow for the inward passage of sodium ions or outward passage of potassium ions are indicated with solid arrows (membrane clock). Channels which allow for the permeability of calcium ions are indicated with dashed arrows (calcium clock). Channels and receptors are colored according to the legend if their presence is statistically significant (p < 0.05). Created with BioRender.

Cluster analysis was performed to categorize expression levels of particular cardiac genes of interest into 4 main groups: 1) cardiac ion channels; 2) cardiac receptors; 3) neural proteins; and 4) transcription factors (Figure 4D). The cardiac genes of interest were selected from a thorough review of published data (21–26), including the most recent “novel” SAN genes identified and published by van Eif et al. (27). and Goodyer et al. (28). The full list of the genes of interest with corresponding FPKM (fragments per kilobase of transcript per million mapped reads) values for the rat is shown in Supplemental Table 3, and the complete heatmap with labeled genes for the rat is shown in Supplemental Figure 6A. Differential expression patterns are observed between the sSAN and iSAN, with significant differences identified for the cardiac receptor group from gene set enrichment analysis (Supplemental Figure 6B), which is consistent with the functional differences observed between the sSAN and iSAN during autonomic stimulation. Specific up-regulated DEGs from each category of the heatmap are shown for the sSAN and iSAN in Figure 4E, with overlapping genes listed in the center column. FPKM values for transcription factors across tissues are shown in Figure 4F, and common ion channels and surface receptors associated with spontaneous diastolic depolarization are shown for the nodal and RA tissues in Figure 5.

### EFFECTS OF PHARMACOLOGICAL INTERVENTION ON HUMAN SAN ACTIVITY.

**FIGURE 6:**
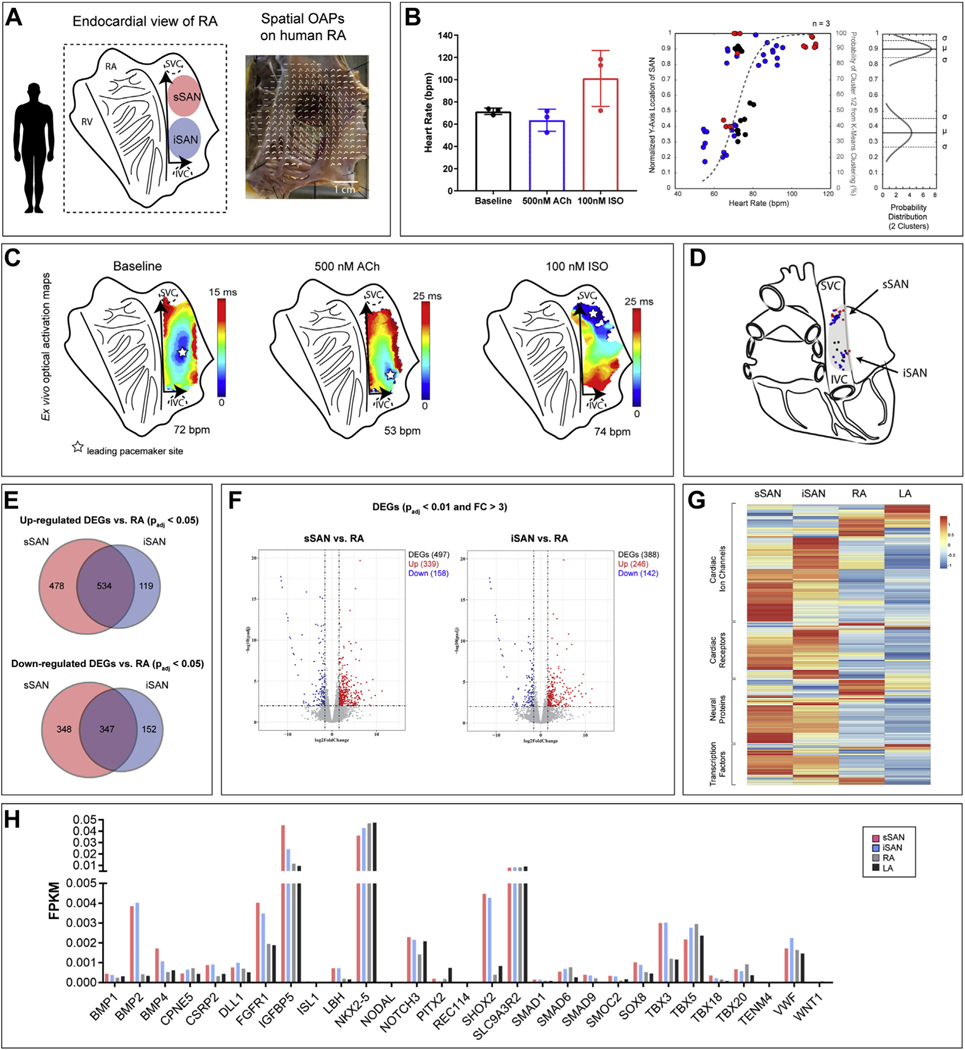
Functional and Molecular Characterization of the Human sSAN and iSAN **(A)** Schematic of the endocardial view of the isolated human RA as well as a representative image of an isolated RA preparation with spatially overlaid OAPs. **(B) (Left)** Intrinsic HRs ex vivo human SANs under baseline conditions and pharmacological stimulation (n = 3). **(Right)** Normalized y-axis locations of leading pacemaker sites under baseline conditions **(black)**, ACh **(blue)**, and ISO **(red)** plotted against corresponding HRs. Points were assigned to 1 of 2 cluster locations (superior or inferior) and fitted with logistic regression curves using k-means clustering analysis **(gray dashed line)** and a normal probability distribution. **(C)** Representative OAPs of an isolated human RA preparation during baseline sinus rhythm, high parasympathetic stimulation (500-nM ACh), and high sympathetic stimulation (100-nM ISO). **(D)** Schematic of the posterior view of the human heart with the 2 spatially distinct pacemakers. **(E)** Venn diagrams depicting the number of up- and down-regulated DEGs for the sSAN and iSAN as compared with the neighboring RA. **(F)** Volcano plots for the sSAN and iSAN as compared with RA. **(G)** Heatmap showing the expression patterns of 4 categories of genes of interest for 4 different tissue regions. Genes within each category were subject to hierarchical clustering. GSEA analysis of the 4 gene sets found no significant differences between sSAN and iSAN in any category. **(H)** Fragments per kilobase of transcript per million mapped reads (FPKM) of cardiac-specific transcription factors across tissues.

Similar to the intact ex vivo rat SAN experiments, we optically mapped intact human RA preparations containing the SAN pacemaking complex (Figure 6A). Here, the average ex vivo human HR at baseline was 71.45 ± 2.91 beats/min, which decreased to 63.52 ± 9.92 beats/min with 500-nM ACh and increased to 101.20 ± 25.25 with 100-nM ISO (n = 3) (Figure 6B). Similar to the rat RA, the spatial distribution of leading pacemaker sites of the human RA fits into 2 binary logistic regression curves (sSAN: 0.90 ± 0.05 AU; iSAN: 0.36 ± 0.09 AU) when plotted against corresponding HR (range 52.50 to 118.40 beats/min). Unlike the rat heart, however, the human heart did not display the same preferential sSAN activity with adrenergic stimulation or iSAN activity with muscarinic stimulation. Nevertheless, complete regional dominance of the iSAN was still identified in situations of low HRs (<66.00 beats/min), while the sSAN dominated at high HRs (>81.00 beats/min) in the human heart. Representative optical activation maps are shown in Figure 6C, and a novel schematic indicating the physiological presence of both a superior and inferior pacemaker region from the compilation of ex vivo leading pacemaker points in the human heart is displayed in Figure 6D.

### RNA SEQUENCING OF HUMAN sSAN AND iSAN.

RNA sequencing was performed on human tissues that were not used for functional experiments in an additional 3 hearts for SAN tissues and 5 hearts for myocardial tissues (Supplemental Table 1). Although there were no DEGs observed between the 2 sSAN and iSAN regions, the human sSAN possessed 4-fold as many up-regulated DEGs and 2-fold as many down-regulated DEGs (p_adj_ < 0.05) than the iSAN when compared with RA genes (Figure 6E). Even with more stringent statistical boundaries (p_adj_ < 0.01, FC > 3), there were still hundreds of DEGs observed for both regions (Figure 6F). Leukocyte migration, positive regulation of cytokine production, and cell chemotaxis were among the most significantly enriched GOs for both the human sSAN and iSAN (Supplemental Figure 7A). In observing a heatmap of genes known to play a role in cardiac function (Figure 4G, Supplemental Figure 8), gene set enrichment analysis found no significant differences between the sSAN and iSAN in any of the 4 gene sets examined for the human (data not shown). The full list of the genes of interest with corresponding FPKM (fragments per kilobase of transcript per million mapped reads) values for the human heart is shown in Supplemental Table 4. Comparisons of common cardiac transcription factors are shown for the human sSAN, iSAN, RA, and left atrium in Figure 6H.

## DISCUSSION

Here, we show that the healthy, denervated ex vivo isolated rat and human RA both exhibit 2 distinct clusters of functional pacemaking activity specifically located near the SVC and IVC—denoted here as the sSAN and iSAN—which preferentially control high and low HRs, respectively (Central Illustration). Previous reports from various species (3,12,29–33) have also demonstrated that leading pacemaker sites can and generally do move toward an inferior location within the SAN with high vagal tone (in addition to activity from the atrioventricular node or atrioventricular ring) (34), but this inferior, or “backup,” pacemaker located near the IVC has not yet been fully characterized. With the application of state-of-the-art optical mapping and novel signal processing techniques on a beat-to-beat basis, we observed a clear phenomenon of 2 distinct RA pacemaking regions over the entire range of SAN-initiated physiological HRs in the healthy rat heart and over a normal range of physiological HRs in the healthy human heart. Additionally, these leading pacemaker sites, sSAN and iSAN, within the pacemaker complex do not appear to show gradual shifting capabilities when the intrinsic HR is altered, but rather they cluster into 2 discrete anatomical regions of automaticity, “jumping” between the 2 sites when HR is altered. Tissue-level RNA sequencing confirmed unique, distinctive mRNA profiles for both the sSAN and iSAN regions, suggesting the ability of the iSAN to behave as its own dominant pacemaker and thus providing a second available pacemaking region within the SAN complex to achieve the full range of physiological HRs when the sSAN is silent.

### DUAL SAN PACEMAKERS IN THE RAT RA.

Although the ex vivo intact healthy rat sSAN was shown to only dominate at normal-to-high physiological HRs, we found that the physically separated iSAN can robustly support almost the entire range of physiological HRs. These findings agree with previously held notions that a “latent” pacemaker can serve as a “dominant” pacemaker when cells with higher automaticity have been suppressed or removed (4), as well as the knowledge that latent pacemaking activity occurs close to the IVC (29). This also sheds new light on the important functionality of the iSAN, a structure that has otherwise only minimally been studied to date.

Interestingly, under failing conditions, only one SAN region remained present. Though SNRT analyses initially suggested that the rat SAN was not detrimentally affected by the severe and aggressive form of left ventricular hypertrophy via ascending aortic constriction, optical mapping identified distinct functional changes of the SAN complex in end-stage heart failure. Though only functionally explored in this paper, the failing rat heart findings emphasize the importance and rationale of 2 distinct pacemaking clusters for normal, healthy hearts to achieve the wide range of physiological HRs.

Relative to the RA, both the sSAN and iSAN in the healthy rat were found to contain a number of cardiac ion channels known to play a predominant role in pacemaking activity (Figures 4 and 5). Specifically, both nodal tissues expressed significantly higher levels of the voltage membrane currents *HCN1*, *HCN4*, *Scn3a* (Nav1.3), and *Kcnk1* (TWIK1). However, neither of the calcium channels known to play a role in nodal activity was found to be relatively higher in either nodes; the 2 L-type calcium channels *Cacna1c* (CaV1.2) and *Cacna1d* (CaV1.3) as well as the 2 T-type calcium channels *Cacna1h* (CaV3.2) and *Cacna1g* (CaV3.1) were significantly higher in the right atria. However, calmodulin (CALM1), which is known to play a role in calcium binding and the regulation of HR, was higher in both nodes respective to the adjacent myocardium.

In terms of unique genetic profiling, the relative density of certain adrenergic receptors and neuronal proteins might explain preferential activation of one SAN region over the other. For instance, only the sSAN displayed statistically higher expression levels of the alpha-2B adrenergic receptor and tyrosine hydroxylase, while only the iSAN expressed high levels of the cholinergic nicotinic receptor, Chrna7. Also interesting is the high number of metabolic markers that were uniquely identified in the sSAN; this confirms the “textbook” or sSAN’s unique ability to recover from severe hypoxic conditions that irreversibly damage the working myocardium (35) and further explains the ability of the healthy rat sSAN in this study to readily recover with high HRs, even after rendered functionally silent by ACh administration. Such findings warrant further investigations.

### DUAL SAN PACEMAKERS IN THE HUMAN RA.

Although the denervated human hearts in this study also displayed 2 spatially different pacemaking regions, preference for sSAN or iSAN initiation did not appear to solely depend on pharmacological intervention; the human sSAN could sometimes initiate in the presence of ACh, and the iSAN could sometimes initiate in the presence of ISO. Likely, this was due to an insufficient washout step which was limited due to the short lifetime of the perfused preparation, leaving residual “opposing” pharmacological stimuli effects in the tissue prior to the onset of the pharmacological stimulus. Additionally, though the human donor hearts taken for optical mapping studies in our lab are prescreened for underlying heart conditions, several other factors (e.g., age, sex, comorbidities, downtime, cardioplegic arrest, etc.) may contribute to heterogeneous functioning of the human SAN (mean human heart age in this study was 51 years). Still, the clear evidence of 2 distinct pacemakers in the human heart support the conclusion of spatial preferential control of high and low physiological HRs.

Tissue-level RNA sequencing data from the human SAN tissues extracted for this study did not identify markedly different levels of pacemaking specific ion channels (e.g., HCN1 or HCN4) or connexins (e.g., Cx40 or Cx43) between either pacemakers or atrial myocardium, indicating significant presence of remnant atrial tissue present in the nodal tissue samples. This is an apparent limitation of tissue-level RNA sequencing for large mammalian hearts, in which only very small spatial samples are used for analysis. Regardless, the high number of SAN-specific developmental transcription factors in both nodal regions support the hypothesis that the large region of tissue between the SVC-IVC axis is derived from the same cells during development, making this entire region capable of pacemaking activity. Future single-cell RNA sequencing studies will be required to address this limitation and further support this hypothesis.

### STUDY LIMITATIONS.

In addition to the novel findings presented here, several limitations are still noted. First, although the presence of only one active pacemaking region was found to be evident in the failing rat heart, our particular left ventricular hypertrophy model of end-stage heart failure did not elicit a bradycardic or sick sinus syndrome response. As a result, it can only be speculated how the sSAN and iSAN adapt under more physiologically relevant SA nodal failing conditions. Future studies should investigate regional SAN activity in a variety of heart failure models and correlate functional findings with cell- or tissue-level gene expression. Second, tissue-level RNA sequencing may have limitations for detailed investigations of SA nodal activity. Although we were able to observe certain similarities between the sSAN and iSAN in comparison with the neighboring myocardium, the manual dissection of these regions for RNA sequencing could only be based on functional findings from other hearts. We observed variability of pacemaking activity both within and across hearts, so the accurate anatomical dissection of true sSAN or iSAN for sequencing could only be assumed. Additionally, large amounts of atrial-specific genetic markers were still evident in the nodal regions, indicating that tissues along the SVC-IVC region contain variable amounts of nodal and myocardial cells. Future studies should employ more specific cell-level RNA sequencing for more representative findings. Lastly, the findings from the intact and cut ex vivo rat SAN may present some results that could not be fully accounted for, as we physically cut through the nodal tissues, myocardial tissues, and areas of ganglionated plexi when separating the sSAN and iSAN. In addition to aberrant pacemaking activity at the edges of the cut tissues, general pathways of neuronal activity between the ganglionated plexi and nodal tissues in the rat heart are still not well understood. Physical separation of the nodal regions from their neighboring ganglionated plexi could have uniquely affected HR results and leading pacemaker site locations.

## CONCLUSIONS

Overall, in addition to contributing to basic biomedical science, the results presented here shed new light on the functionality of the heterogeneous pacemaking complex and offer a more complete understanding of the origin of the heartbeat. It is theorized that more than one distinct pacemaker site allows for the coordinated and integrated firing of nodal tissue to achieve a range of physiological HRs, particularly when these HRs change rapidly. These findings are particularly important for elucidating, and thus potentially clinically overcoming, pathological conditions such as SAN dysfunction, ectopic beats during atrial arrhythmias, junctional rhythms, and HR variability related to sudden cardiac death. Future studies should look at other potential pacemaker regions located near orifices of the coronary sinus and pulmonary veins using single-cell RNA sequencing and single-cell electrophysiology techniques. These studies should be also carried out in human hearts with a history of atrial arrhythmias, which tend to originate from these regions.

## Supplementary Material

mmc1

## Figures and Tables

**CENTRAL ILLUSTRATION:**
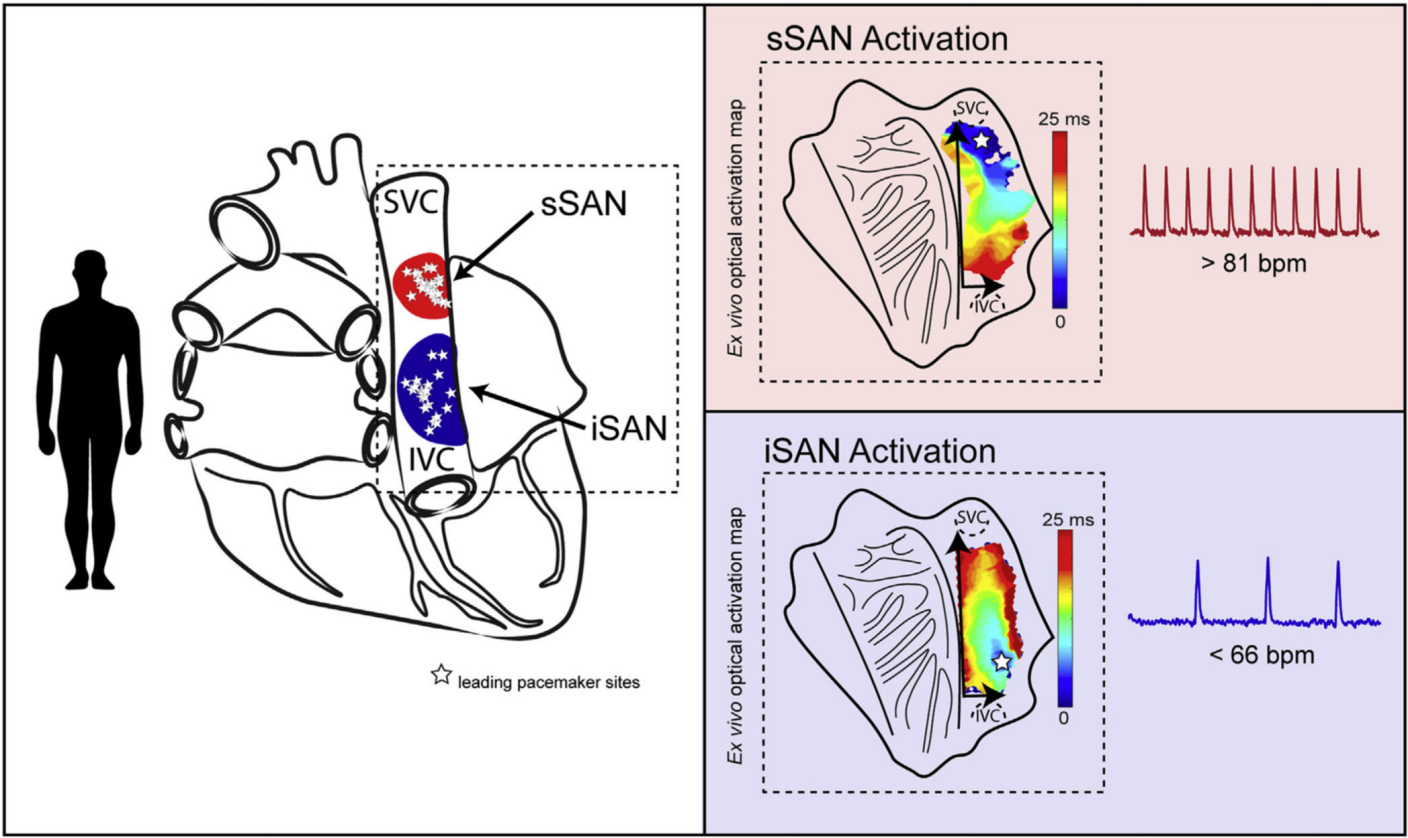
Sinus Rhythm Is Driven by 2 Distinct Pacemaking Regions in the Human Heart The normal sinus rhythm is driven by 2 distinct pacemaking regions in the human heart: the superior sinoatrial node (sSAN) and inferior SAN (iSAN), which are located near the superior vena cava (SVC) and inferior vena cava (IVC), respectively. The sSAN and iSAN preferentially control high and low physiological heart rates, respectively. **(Left)** Ex vivo leading pacemaker sites during normal sinus rhythm across a range of heart rates in 3 human hearts and **(right)** representative optical activation maps for beats during fast sinus rhythm **(top right)** and slow sinus rhythm **(bottom right)**. SVC = superior vena cava; IVC = inferior vena cava; sSAN = superior sinoatrial node; iSAN = inferior sinoatrial node.

## PERSPECTIVES

### COMPETENCY IN MEDICAL KNOWLEDGE:

The findings reported here shed light on the presence of 2 distinct RA pacemakers in the healthy human heart that dominate at distinct ranges of HRs. Identified in the human heart and fully characterized in the rat heart, these pacemakers are preferentially located at the 2 orifices of the venae cavae and abruptly switch their dominance to maintain stable physiological rates. Such novel findings may help clarify previous experimental and clinical observations of a P-wave inversion and wandering pacemaker. The results reported here also indicate that only 1 of these 2 pacemakers remain functional during end-stage heart failure, and SNRT evaluation is not a sufficient tool for elucidating the number of active pacemakers in the heart.

### TRANSLATIONAL OUTLOOK:

High-resolution ex vivo optical mapping affords the ability to deeply investigate changes in pacemaking activity in both healthy and failing hearts on a beat-to-beat basis. With the novel understanding that there are 2 distinct, functionally active sinoatrial nodal pacemakers in the mammalian heart, it will be imperative to further investigate the physiological rationale of their spatial dominance, especially during pathogenesis of heart failure. In addition to the up- or down-regulation of certain cardiac ion channels, cardiac receptors, neural proteins, and transcription factors within each of these nodal regions, it will be worth investigating other factors such as cell-to-cell coupling dynamics, adrenergic and cholinergic sensitivity, and cardiac remodeling of SA nodal tissue during heart failure to answer current unknowns about their spatial and temporal functionalities. Although tissue-level studies such as this are imperative for identifying broad and clinically relevant trends, future studies should examine cell- and protein-level contributions of pacemaking activity to clarify such findings. Deeper exploration into other regions of known ectopic activity at the orifices of major vessels (e.g., pulmonary veins) will also be crucial for providing clinicians with deeper insights into the inner workings of arrhythmogenicactivity.

## ABBREVIATIONS AND ACRONYMS

ACh: acetylcholine
DEG: differentially expressed gene
HR: heart rate
iSAN: inferior sinoatrial node
ISO: isoproterenol
IVC: inferior vena cava
OAP: optical action potential
RA: right atrium
SAN: sinoatrial node
sSAN: superior sinoatrial node
SNRT: sinus node recovery time
SVC: superior vena cava

## References

[1] Schuessler RB, Boineau JP, Bromberg BI. Origin of the sinus impulse. J Cardiovasc Electrophysiol 1996;7:263–74. 8867301 10.1111/j.1540-8167.1996.tb00524.x

[2] Boyett MR, Honjo H, Kodama I. The sinoatrial node, a heterogeneous pacemaker structure. Cardiovasc Res 2000;47:658–87. 10974216 10.1016/s0008-6363(00)00135-8

[3] Glukhov AV, Fedorov VV, Anderson ME, Mohler PJ, Efimov IR. Functional anatomy of the murine sinus node: High-resolution optical mapping of ankyrin-B heterozygous mice. Am J Physiol Heart Circ Physiol 2010;299:H482–91. 20525877 10.1152/ajpheart.00756.2009 PMC2930390

[4] Lu HH. Shifts in pacemaker dominance within the sinoatrial region of cat and rabbit hearts resulting from increase of extracellular potassium. Circ Res 1970;26:339–46. 5415862 10.1161/01.res.26.3.339

[5] Mackaay AJC, Op’t Hof T, Bleeker WK, Jongsma HJ, Bouman LN. Interaction of adrenaline and acetylcholine on cardiac pacemaker function. Functional inhomogeneity of the rabbit sinus node. J Pharmacol Exp Ther 1980;214:417–22. 7391985

[6] Shibata N, Inada S, Mitsui K, Pacemaker shift in the rabbit sinoatrial node in response to vagal nerve stimulation. Exp Physiol 2001;86: 177–84. 11429632 10.1113/eph8602100

[7] Boineau JP, Schuessler RB, Roeske WR, Autry LJ, Miller CB, Wylds AC. Quantitative relation between sites of atrial impulse origin and cycle length. Am J Physiol Heart Circ Physiol 1983; 245:H781–9. 10.1152/ajpheart.1983.245.5.H781 6638200

[8] Fedorov VV, Glukhov AV, Chang R, Optical mapping of the isolated coronary-perfused human sinus node. J Am Coll Cardiol 2010;56:1386–94. 20946995 10.1016/j.jacc.2010.03.098 PMC3008584

[9] Opthof T, VanGinneken ACG, Bouman LN, Jongsma HJ. The intrinsic cycle length in small pieces isolated from the rabbit sinoatrial node. J Mol Cell Cardiol 1987;19:923–34. 3430642 10.1016/s0022-2828(87)80621-1

[10] Monfredi O, Dobrzynski H, Mondal T, Boyett MR, Morris GM. The anatomy and physiology of the sinoatrial node-A contemporary review. Pacing Clin Electrophysiol 2010;33: 1392–406. 20946278 10.1111/j.1540-8159.2010.02838.x

[11] Boukens BJ, Efimov IR. A century of optocardiography. IEEE Rev Biomed Eng 2014;7: 115–25. 24158521 10.1109/RBME.2013.2286296 PMC4521770

[12] Efimov IR, Fedorov VV, Joung B, Lin SF. Mapping cardiac pacemaker circuits: Methodological puzzles of the sinoatrial node optical mapping. Circ Res 2010;106:255–71. 20133911 10.1161/CIRCRESAHA.109.209841 PMC2818830

[13] Yanni J, Tellez JO, Sutyagin PV, Boyett MR, Dobrzynski H. Structural remodelling of the sinoatrial node in obese old rats. J Mol Cell Cardiol 2010;48:653–62. 19729016 10.1016/j.yjmcc.2009.08.023 PMC2845824

[14] Buchman TG, Stein PK, Goldstein B. Heart rate variability in critical illness and critical care. Curr Opin Crit Care 2002;8:311–5. 12386491 10.1097/00075198-200208000-00007

[15] Nabipour A. Comparative histological structure of the sinus node in mammals. Anim Sci 2012;36: 463–9.

[16] Kim D, Langmead B, Salzberg SL. HISAT: A fast spliced aligner with low memory requirements. Nat Methods 2015;12:357–60. 25751142 10.1038/nmeth.3317 PMC4655817

[17] Dobin A, Davis CA, Schlesinger F, STAR: Ultrafast universal RNA-seq aligner. Bioinformatics 2013;29:15–21. 23104886 10.1093/bioinformatics/bts635 PMC3530905

[18] Liao Y, Smyth GK, Shi W. FeatureCounts: An efficient general purpose program for assigning sequence reads to genomic features. Bioinformatics 2014;30:923–30. 24227677 10.1093/bioinformatics/btt656

[19] Love MI, Huber W, Anders S. Moderated estimation of fold change and dispersion for RNA-seq data with DESeq2. Genome Biol 2014;15:550. 25516281 10.1186/s13059-014-0550-8 PMC4302049

[20] Yu G, Wang LG, Han Y, He QY. ClusterProfiler: An R package for comparing biological themes among gene clusters. OMICS 2012;16:284–7. 22455463 10.1089/omi.2011.0118 PMC3339379

[21] Ferdous Z, Queshi MA, Jayaprakash P, Different profile of mRNA expression in sinoatrial node from streptozotocin-induced diabetic rat. PLoS One 2016;11:e0153934. 27096430 10.1371/journal.pone.0153934 PMC4838258

[22] Bult CJ, Blake JA, Smith CL, Kadin JA, Richardson JE. Mouse Genome Database Group. Mouse Genome Database (MGD) 2019. Nucleic Acids Res 2019;47:D801–6. 30407599 10.1093/nar/gky1056 PMC6323923

[23] Medical College of Wisconsin. Rat Genome Database. Available at: https://rgd.mcw.edu/. Accessed April 15, 2020.

[24] van Weerd JH, Christoffels VM. The formation and function of the cardiac conduction system. Development 2016;143:197–210. 26786210 10.1242/dev.124883

[25] Park D, Fishman G. Basic science for clinicians: The cardiac conduction system. Circulation 2011; 123:904–15. 21357845 10.1161/CIRCULATIONAHA.110.942284 PMC3064561

[26] van Eif VWW, Devalla HD, Boink GJJ, Christoffels VM. Transcriptional regulation of the cardiac conduction system. Nat Rev Cardiol 2018; 15:617–30. 29875439 10.1038/s41569-018-0031-y

[27] van Eif VWW, Stefanovic S, van Duijvenboden K, Transcriptome analysis of mouse and human sinoatrial node cells reveals a conserved genetic program. Development 2019; 146:dev173161. 10.1242/dev.173161 30936179

[28] Goodyer WR, Beyersdorf BM, Paik DT, Transcriptomic profiling of the developing cardiac conduction system at single-cell resolution. Circ Res 2019;125:379–97. 31284824 10.1161/CIRCRESAHA.118.314578 PMC6675655

[29] Morris GM, D’Souza A, Dobrzynski H, Characterization of a right atrial subsidiary pacemaker and acceleration of the pacing rate by HCN over-expression. Cardiovasc Res 2013; 100:160–9. 23787003 10.1093/cvr/cvt164

[30] Lang D, Petrov V, Lou Q, Osipov G, Efimov IR. Spatiotemporal control of heart rate in a rabbit heart. J Electrocardiol 2011;44:626–34. 21937057 10.1016/j.jelectrocard.2011.08.010 PMC3220935

[31] Ashton JL, Burton RAB, Bub G, Smaill BH, Montgomery JM. Synaptic plasticity in cardiac innervation and its potential role in atrial fibrillation. Front Physiol 2018;9:240. 29615932 10.3389/fphys.2018.00240 PMC5869186

[32] Ashton JL, Trew ML, LeGrice IJ, Shift of leading pacemaker site during reflex vagal stimulation and altered electrical source-to-sink balance. J Physiol 2019;597:3297–313. 31087820 10.1113/JP276876

[33] Boineau JP, Canavan TE, Schuessler RB, Cain ME, Corr PB, Cox JL. Demonstration of a widely distributed atrial pacemaker complex in the human heart. Circulation 1988;77: 1221–37. 3370764 10.1161/01.cir.77.6.1221

[34] Logantha SJRJ, Kharche SR, Zhang Y, Sinus node-like pacemaker mechanisms regulate ectopic pacemaker activity in the adult rat atrioventricular ring. Sci Rep 2019;9:11781. 31409881 10.1038/s41598-019-48276-0 PMC6692414

[35] Gu J, Grijalva SI, Fernandez N, Kim E, Foster DB, Cho HC. Induced cardiac pacemaker cells survive metabolic stress owing to their low metabolic demand. Exp Mol Med 2019;51:1–12. 10.1038/s12276-019-0303-6 PMC6802647 31519870

