# Supplementary material for: Evidence of Superior and Inferior Sinoatrial Nodes in the Mammalian Heart
Source: JACC Clin Electrophysiol. Author manuscript; Available in PMC 2020 Dec 29. (PMC7770336; doi:10.1016/j.jacep.2020.09.012)
Supplement: mmc1 [file NIHMS1630633-supplement-mmc1.docx]

**SUPPLEMENTARY MATERIAL

Supplementary Figure 1:** Spatial distribution of leading pacemaker sites in the normal, intact rat SAN plotted along a normalized y-axis between the SVC and IVC and a scaled x-axis in millimeters (n = 8). Colors correspond to treatment condition (black: baseline, blue: 100 μM ACh, red: 500 nM ISO).


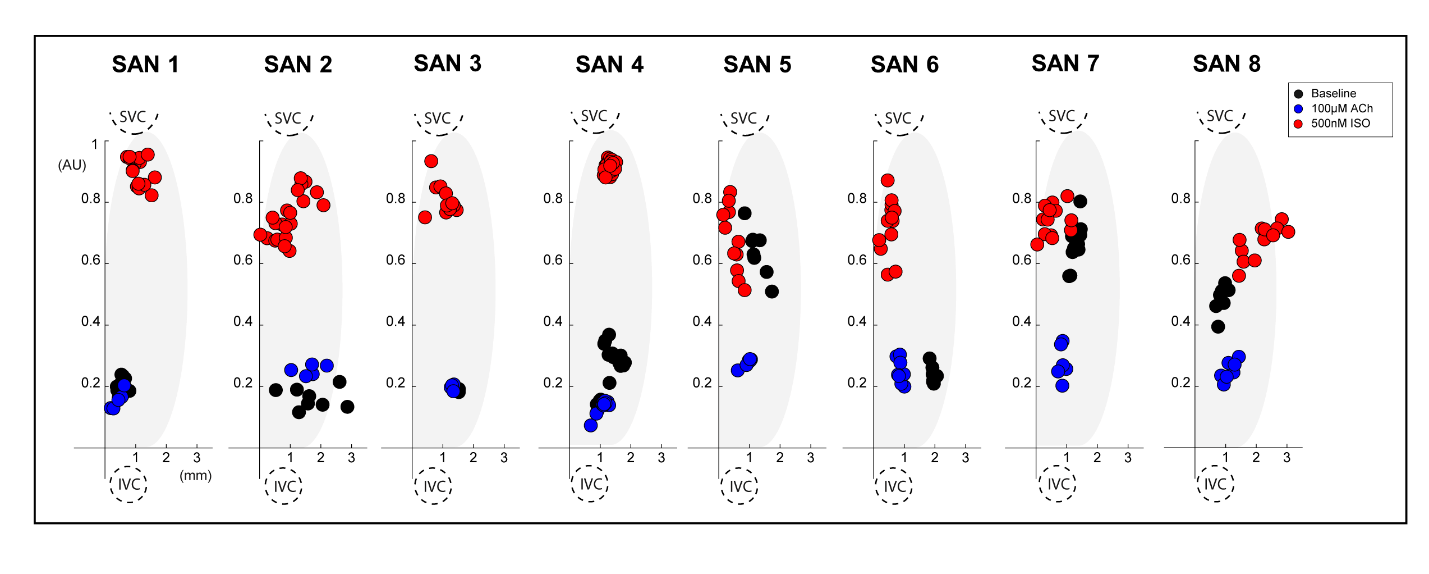


**Supplementary Figure 2:** Spatial distribution of leading pacemaker sites before and after surgical separation of the rat SAN (n = 5). *Top:* Intact SAN preparations with leading pacemaker sites plotted during baseline conditions. *Bottom:* Surgically cut SAN preparations with leading pacemaker sites plotted during baseline conditions (black) and exposure to pharmacological stimulation (blue: 100 μM ACh, red: 500 nM ISO).


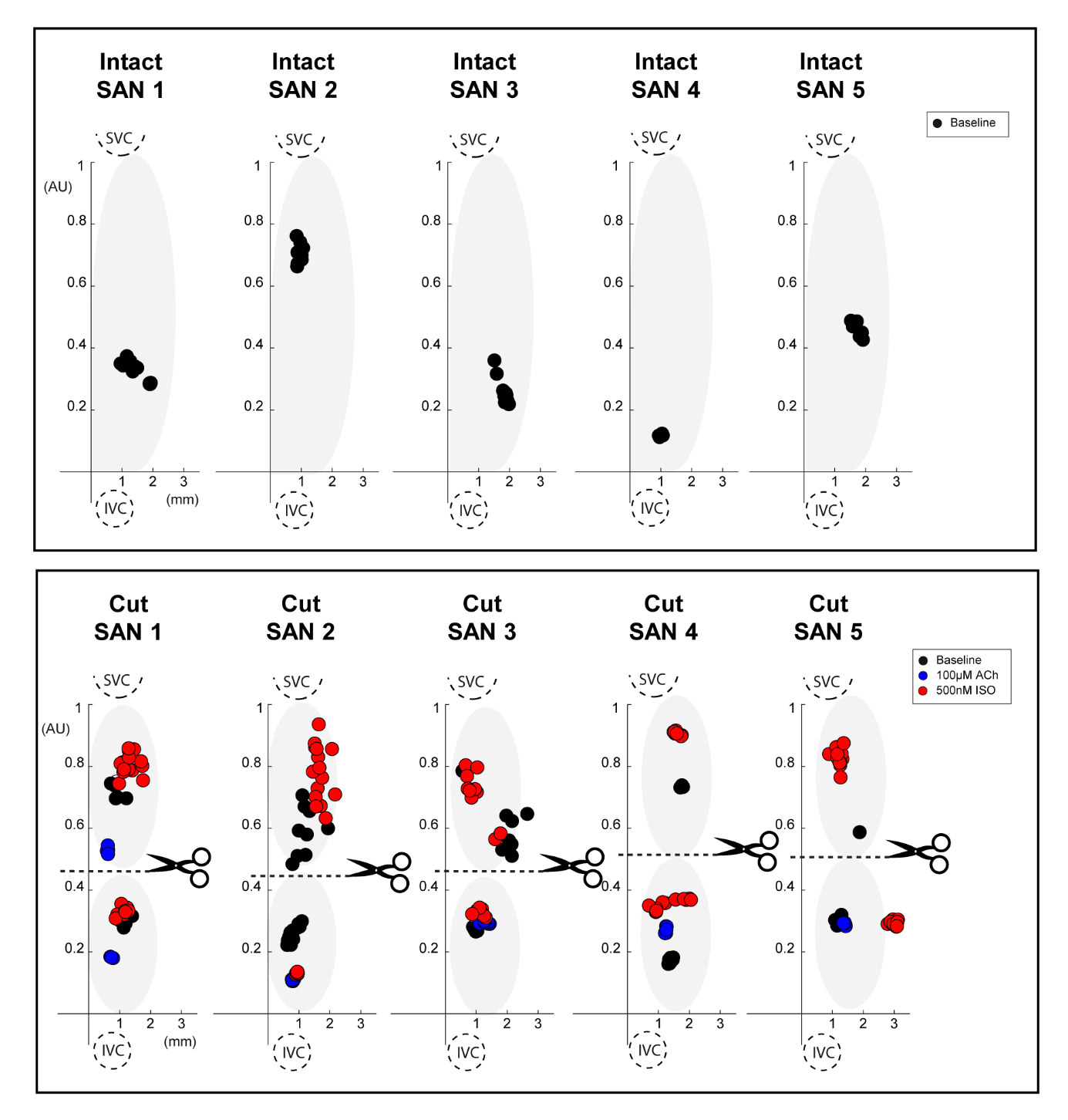


**Supplementary Figure 3: a)** Heatmaps of cardiac genes of interest for individual tissue regions of the rat heart: sSAN, iSAN, RA, and LA. **b)** GSEA analysis between the sSAN and iSAN showing significant differences identified for the cardiac receptor group.


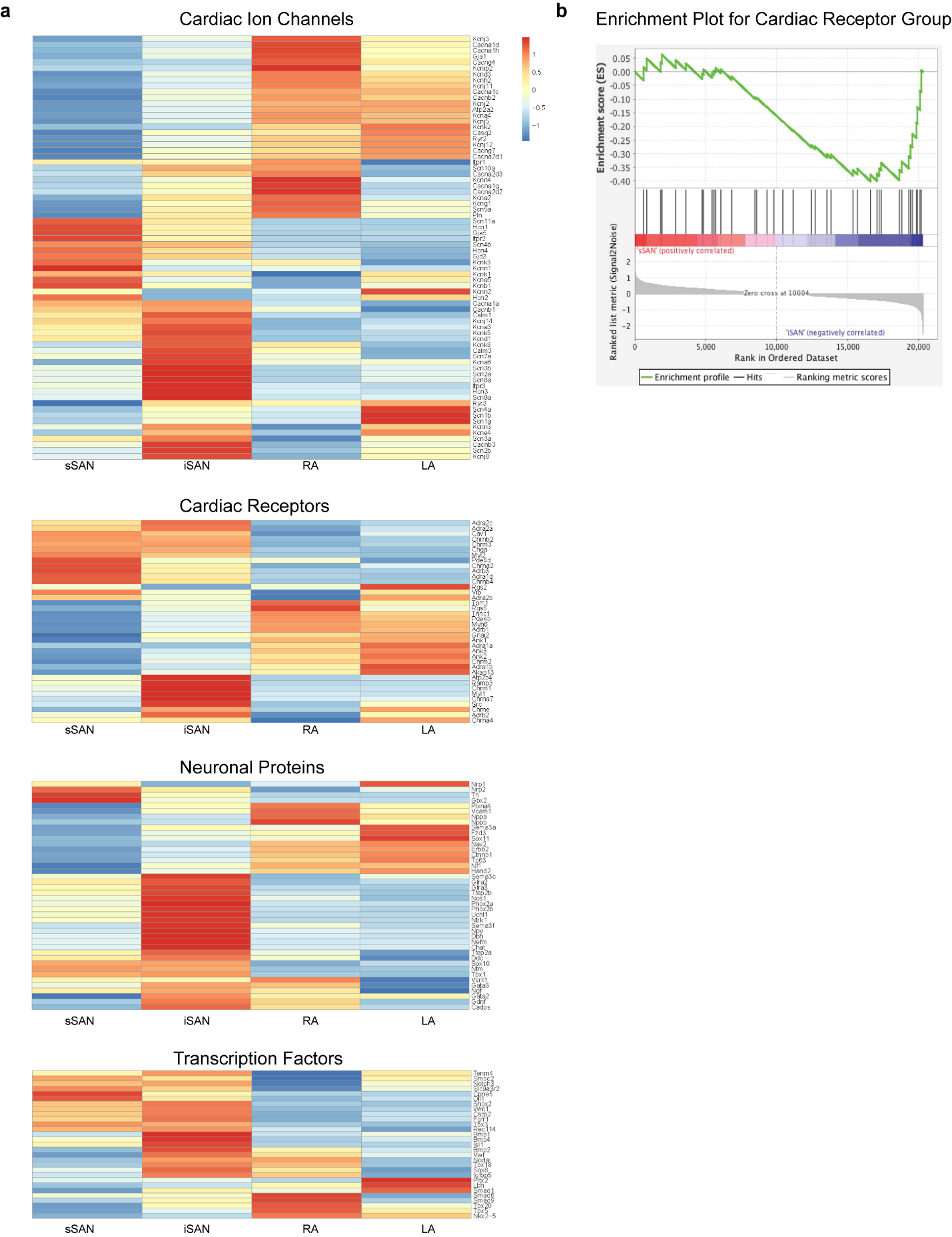


**Supplementary Figure 4:** Characterization of up-regulated DEGs in the human sSAN and iSAN as compared to the RA. **a)** GO analysis with stringent statistical conditioning (padj < 0.01 and FC > 3). **b)** List of up-regulated cardiac DEGs present in the sSAN and iSAN (statistical conditioning padj < 0.05).


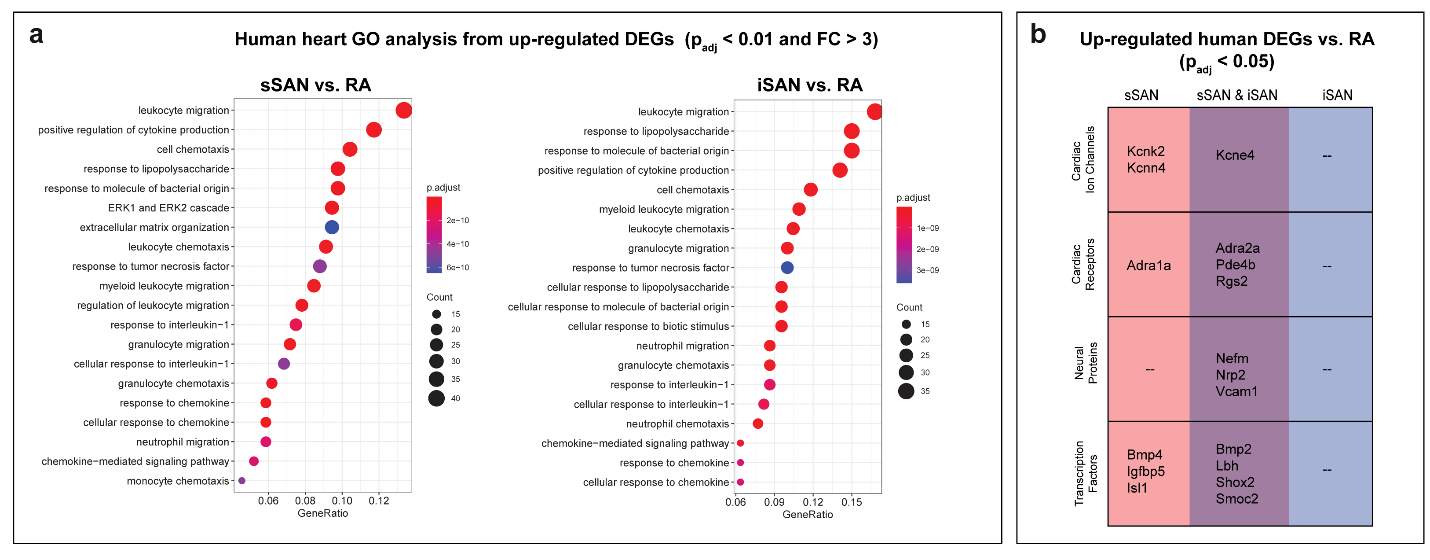


**Supplementary Figure 5:** Heatmaps of cardiac genes of interest for individual tissue regions of the human heart: sSAN, iSAN, RA, and LA.


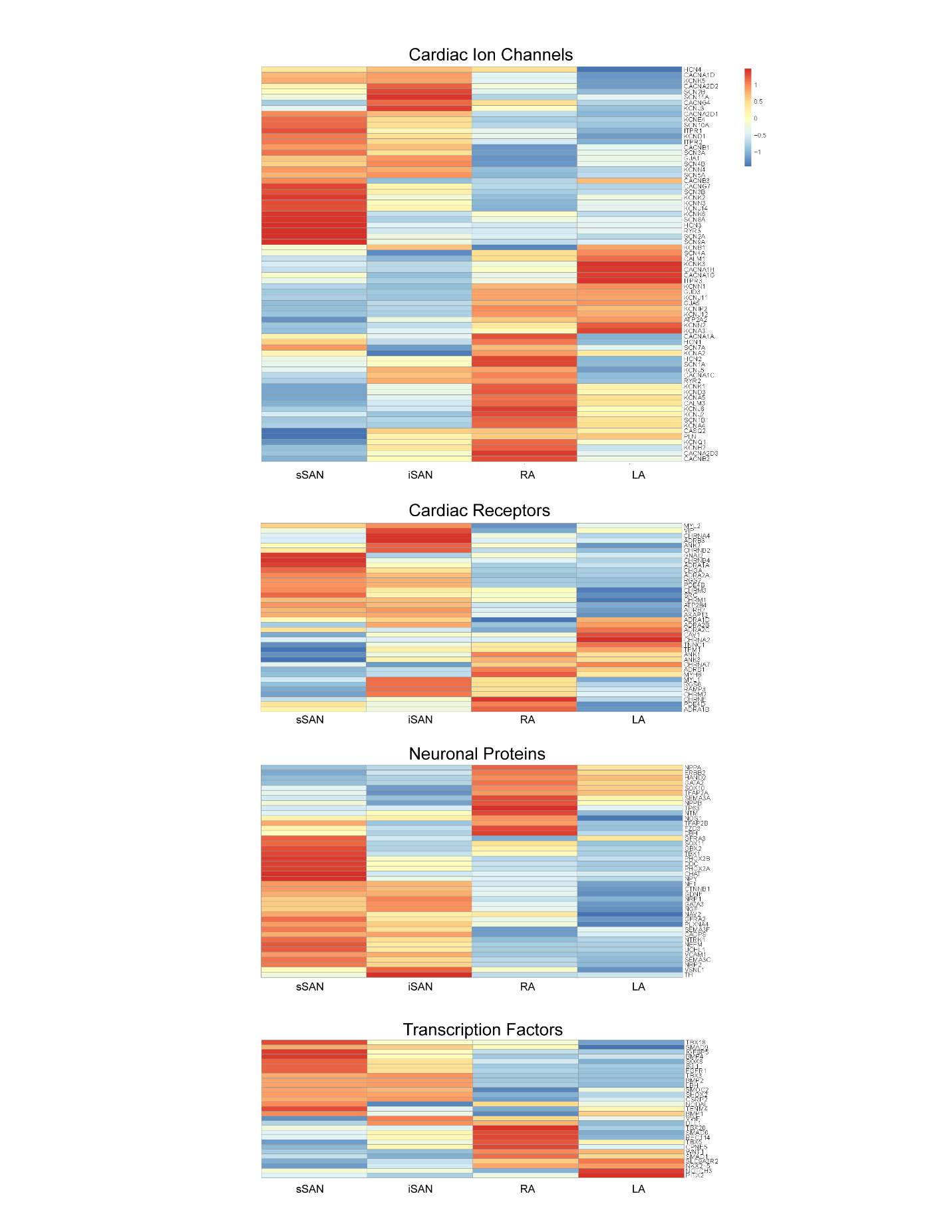


**Supplementary Figure 6: a)** Representative images of healthy and failing, Langendorff-perfused rat hearts. **b)** *In vivo* heart rates of healthy rats over time. **c)** *In vivo* heart rates of failing rats over time.

***
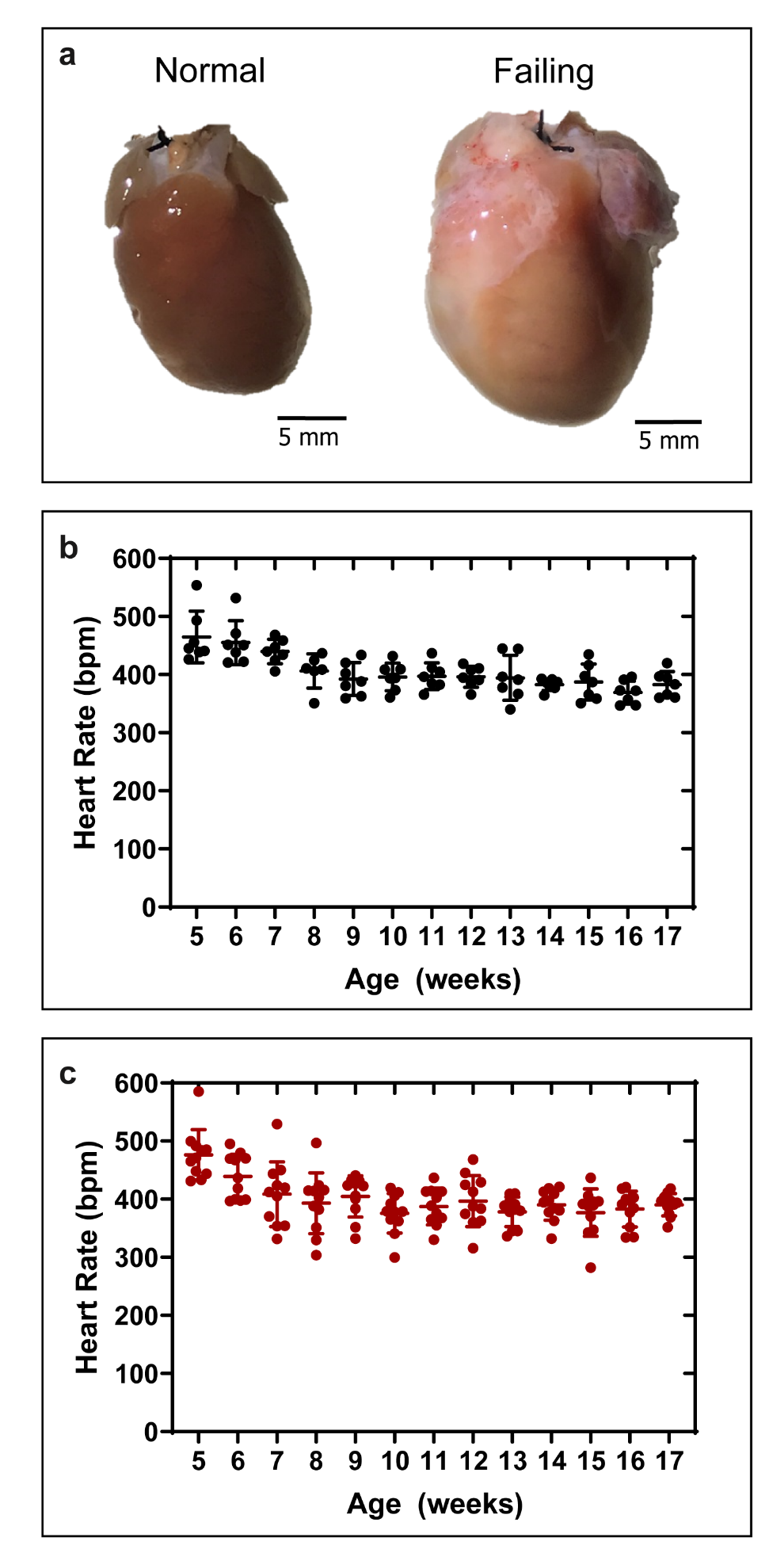
***

**Supplementary Figure 7:** SNRT values of normal/healthy and failing rat hearts. **a)** Process of measuring SNRT values from a stimulus protocol. **b)** All reported SNRT values replotted against heart rate and fit with nonlinear regression analysis. SNRT values for increasing dosages of **c)** ACh and **d)** ISO. No statistical differences were observed between the normal and failing rat heart.


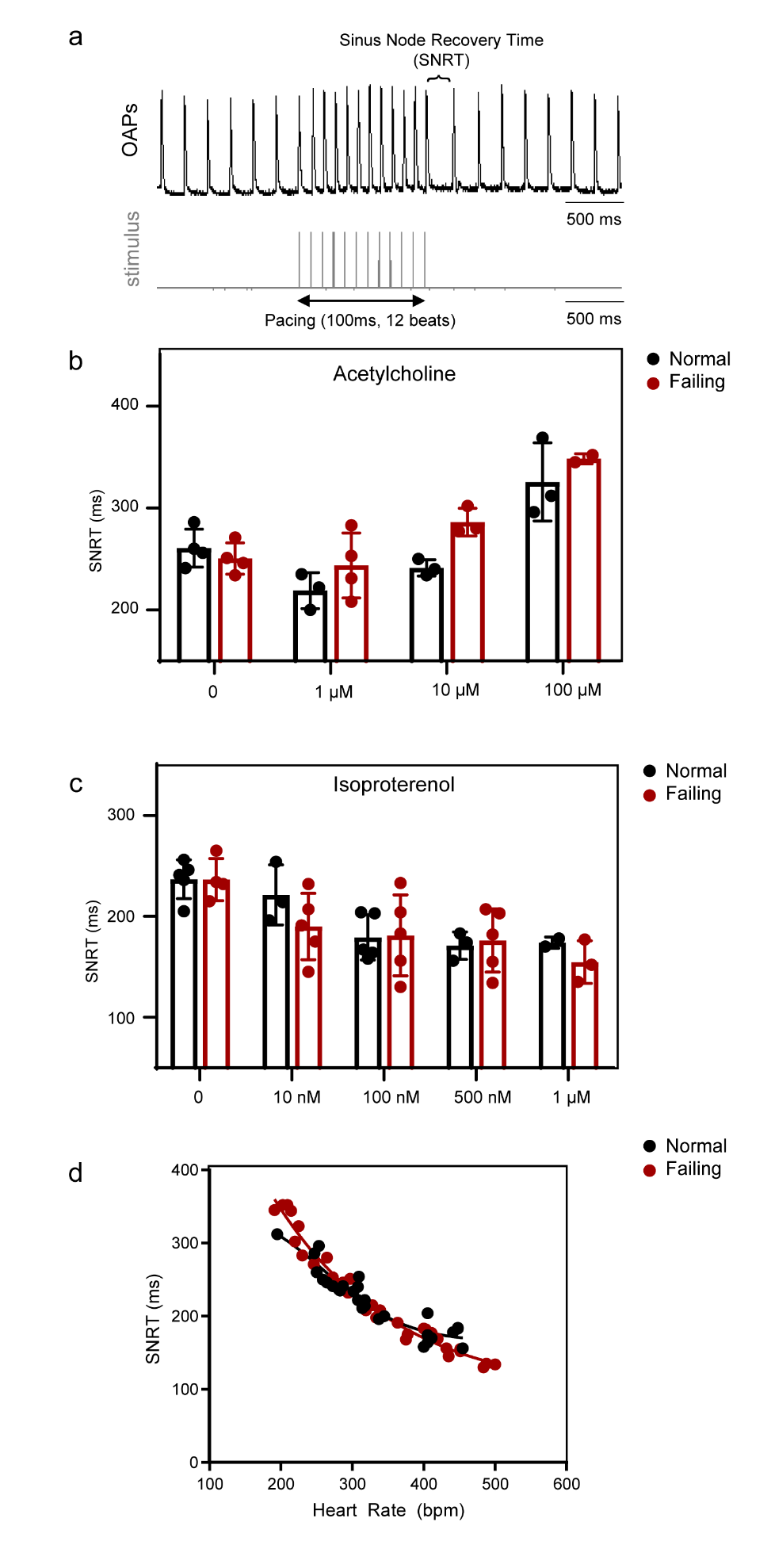


**Supplementary Figure 8:** Spatial distribution of leading pacemaker sites in the SAN of the failing rat heart, plotted along a normalized y-axis between the SVC and IVC and a scaled x-axis in millimeters (n = 6). Colors correspond to treatment condition (black: baseline, blue: 100 μM ACh, red: 500 nM ISO).


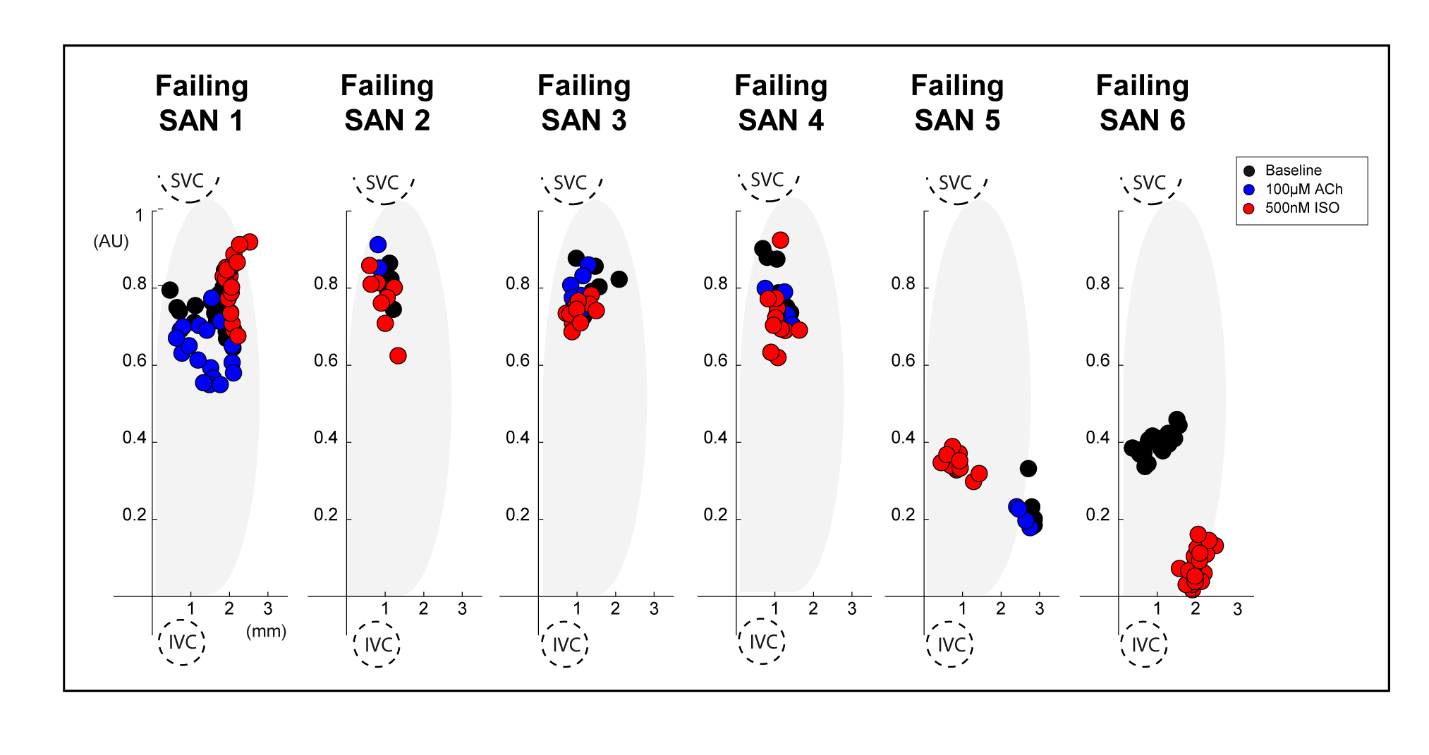


**Supplementary Table 1:** Donor human heart information (LVEF: left ventricular ejection fraction; BMI: body mass index; CVA: cerebrovascular accident).

| **Type of Study** | **Gender** | **Age** | **Cause of Death** | **LVEF (%)** | **BMI** | **Downtime** | **Tissue Procured** |
| --- | --- | --- | --- | --- | --- | --- | --- |
| Functional | F | 37 | Head Trauma | N/A | 25.1 | 15 min | Entire SAN |
| Functional | F | 59 | Anoxia | 45 | 26 | 3 min | Entire SAN |
| Functional | F | 58 | Anoxia | N/A | 16.1 | 6 min | Entire SAN |
| Molecular | F | 46 | CVA/Stroke | 55 | 28.7 | None | sSAN and iSAN |
| Molecular | F | 56 | Anoxia | N/A | 38.9 | 5-10 min | sSAN and iSAN |
| Molecular | F | 71 | Anoxia | 43 | 26.6 | 45 min | sSAN and iSAN |
| Molecular | F | 26 | Infectious Disease | 60-65 | 36.1 | None | RA and LA |
| Molecular | F | 35 | CVA/Stroke | 65 | 31.5 | None | RA |
| Molecular | F | 36 | CVA/Stroke | N/A | 31.7 | None | RA and LA |
| Molecular | M | 26 | Anoxia | 65 | 26.4 | N/A | RA and LA |
| Molecular | M | 34 | Anoxia | 65 | 38.2 | 20 min | LA |

**Supplementary Table 2:** Average FPKM values of the cardiac genes of interest for the rat heart and associated padj values between groups.

**Cardiac Ion Channels**

| **Gene Name** | **Protein** | **sSAN** | **iSAN** | **RA** | **LA** | **LV** | **padj (sSAN vs iSAN)** | **padj (sSAN vs RA)** | **padj (iSAN vs RA)** | **padj (sSAN vs LA)** | **padj (sSAN vs LV)** | **padj (iSAN vs LA)** | **padj (iSAN vs LV)** |
| --- | --- | --- | --- | --- | --- | --- | --- | --- | --- | --- | --- | --- | --- |
| Atp2a2 | SERCA2A | 1568.2226 | 2109.1456 | 2931.0931 | 2900.2031 | 2115.0404 | 0.7499 | 0.0003 | 0.0001 | 0.0003 | 0.0082 | 0.0002 | 0.0014 |
| Cacna1a | Cav2.1 | 1.8391 | 1.9255 | 1.0889 | 0.8017 | 0.7880 | 0.8795 | 0.0010 | 0.0001 | 0.0000 | 0.0255 | 0.0000 | 0.0026 |
| Cacna1c | Cav1.2 | 7.7485 | 11.5016 | 15.3204 | 14.9687 | 14.7354 | 0.3054 | 0.0000 | 0.0063 | 0.0000 | 0.0000 | 0.0106 | 0.0000 |
| Cacna1d | Cav1.3 | 2.1613 | 2.8569 | 5.8618 | 3.9612 | 0.1067 | 0.8728 | 0.0000 | 0.0000 | 0.0167 | 0.0000 | 0.0907 | 0.0000 |
| Cacna1g | Cav3.1 | 15.7716 | 23.1200 | 34.8689 | 15.8276 | 0.7316 | 0.6393 | 0.0005 | 0.0000 | 0.7669 | 0.0000 | 0.0010 | 0.0000 |
| Cacna1h | Cav3.2 | 3.2008 | 4.6428 | 8.2055 | 5.3530 | 1.0545 | 0.8113 | 0.0029 | 0.0000 | 0.1925 | 0.0511 | 0.3162 | 0.0000 |
| Cacna2d1 | Cavα2δ1 | 9.6513 | 16.3678 | 20.7585 | 22.2219 | 8.2979 | 0.2556 | 0.0000 | 0.0346 | 0.0000 | 0.6904 | 0.0055 | 0.1255 |
| Cacna2d2 | Cavα2δ2 | 20.1127 | 26.5468 | 43.9648 | 19.1820 | 6.4697 | 0.8627 | 0.0008 | 0.0001 | 0.6540 | 0.0034 | 0.0431 | 0.0000 |
| Cacna2d3 | Cavα2δ3 | 2.1637 | 3.1362 | 3.5107 | 1.8632 | 0.0565 | 0.7239 | 0.0993 | 0.5797 | 0.3909 | 0.0000 | 0.0044 | 0.0000 |
| Cacnb1 | Cavβ1 | 0.8061 | 0.8556 | 0.6404 | 0.3947 | 0.1206 | 0.9496 | 0.2652 | 0.4759 | 0.0054 | 0.0007 | 0.0236 | 0.0016 |
| Cacnb2 | Cavβ2 | 12.9439 | 18.3177 | 23.5588 | 22.1062 | 24.3995 | 0.6686 | 0.0019 | 0.0764 | 0.0064 | 0.0000 | 0.1792 | 0.0000 |
| Cacnb3 | Cavβ3 | 1.8564 | 2.6595 | 1.4418 | 1.8708 | 0.7962 | 0.6469 | 0.0562 | 0.0005 | 0.7058 | 0.0640 | 0.0933 | 0.0022 |
| Cacng4 | Cavγ4 | 0.8129 | 0.8452 | 1.4077 | 1.1522 | 0.7062 | 0.9313 | 0.0132 | 0.0504 | 0.5495 | 0.7440 | 0.4794 | 0.6032 |
| Cacng7 | Cavγ7 | 0.9899 | 1.1563 | 1.2389 | 1.2989 | 0.5559 | 0.9769 | 0.8074 | 0.9078 | 0.7093 | 0.6541 | 0.8118 | 0.5300 |
| Calm1 | Calm1 | 215.7176 | 238.0546 | 191.0577 | 184.8401 | 168.5449 | 0.7957 | 0.0001 | 0.0002 | 0.0000 | 0.9371 | 0.0000 | 0.5838 |
| Calm3 | Calm3 | 80.3610 | 92.4221 | 83.6242 | 75.3588 | 80.3317 | 0.9989 | 0.6101 | 0.1986 | 0.1349 | 0.1340 | 0.0005 | 0.0030 |
| Casq2 | Casq2 | 303.6929 | 405.9169 | 431.8703 | 487.2037 | 385.2062 | 0.7570 | 0.1312 | 0.5489 | 0.0152 | 0.0174 | 0.0497 | 0.0030 |
| Gja1 | Cx43 | 115.1344 | 134.1806 | 175.1215 | 144.4395 | 126.9160 | 0.9882 | 0.0003 | 0.0011 | 0.2768 | 0.0017 | 0.3896 | 0.0003 |
| Gja5 | Cx40 | 6.4763 | 3.7762 | 1.3930 | 1.6130 | 2.3822 | 0.1269 | 0.0000 | 0.0000 | 0.0000 | 0.0121 | 0.0002 | 0.8891 |
| Gjd3 | Cx30.2 | 3.6111 | 2.9334 | 1.3262 | 0.8344 | 0.4887 | 0.7028 | 0.0001 | 0.0166 | 0.0000 | 0.0001 | 0.0001 | 0.0037 |
| Hcn1 | HCN1 | 1.4405 | 0.6869 | 0.0559 | 0.0080 | 0.0000 | 0.1748 | 0.0000 | 0.0000 | 0.0000 | 0.0000 | 0.0000 | 0.0000 |
| Hcn2 | HCN2 | 5.0825 | 4.4300 | 4.5312 | 4.8952 | 70.5678 | 0.5873 | 0.2119 | 0.9108 | 0.3785 | 0.0000 | 0.6445 | 0.0000 |
| Hcn3 | HCN3 | 0.0036 | 0.0330 | 0.0020 | 0.0000 | 0.0079 | NA | NA | 0.2172 | NA | NA | 0.1932 | 0.6307 |
| Hcn4 | HCN4 | 15.9008 | 11.2801 | 3.6610 | 1.0438 | 2.0174 | 0.4099 | 0.0000 | 0.0000 | 0.0000 | 0.0000 | 0.0000 | 0.0000 |
| Itpr1 | IP3R1 | 16.1416 | 16.0113 | 17.0222 | 13.9778 | 3.5430 | 0.7864 | 0.7591 | 0.6535 | 0.2036 | 0.0000 | 0.4748 | 0.0000 |
| Itpr2 | IP3R2 | 10.0855 | 6.8399 | 4.2355 | 4.3340 | 2.3649 | 0.7499 | 0.0520 | 0.0855 | 0.0587 | 0.1338 | 0.2790 | 0.1951 |
| Itpr3 | IP3R3 | 2.3394 | 3.4600 | 2.1258 | 1.9983 | 1.3373 | 0.4685 | 0.2476 | 0.0005 | 0.1069 | 0.1857 | 0.0000 | 0.0003 |
| Kcna2 | Kv1.2 | 17.9631 | 30.4116 | 36.2632 | 22.1091 | 5.4814 | 0.4294 | 0.0005 | 0.3519 | 0.7350 | 0.0006 | 0.2231 | 0.0000 |
| Kcna3 | Kv1.3 | 0.1415 | 0.1717 | 0.1044 | 0.1185 | 0.1045 | 0.9769 | 0.4685 | 0.4592 | 0.6336 | 0.9734 | 0.6086 | 0.9397 |
| Kcna4 | Kv1.4 | 1.0993 | 1.6037 | 2.5369 | 2.3779 | 1.3538 | 0.7499 | 0.0009 | 0.0026 | 0.0017 | 0.2320 | 0.0057 | 0.3941 |
| Kcna5 | Kv1.5 | 6.9625 | 4.7642 | 3.8801 | 5.6708 | 4.3701 | 0.1228 | 0.0058 | 0.6101 | 0.0344 | 0.5002 | 0.3932 | 0.2559 |
| Kcna6 | Kv1.6 | 0.0433 | 0.1575 | 0.0842 | 0.0882 | 0.0114 | 0.3499 | 0.4473 | 0.2888 | 0.4130 | 0.5453 | 0.3626 | 0.0131 |
| Kcnb1 | Kv2.1 | 6.8293 | 6.0029 | 5.2493 | 5.8126 | 7.3742 | 0.5446 | 0.0487 | 0.5188 | 0.1620 | 0.2208 | 0.9682 | 0.0000 |
| Kcnd1 | Kv4.1 | 0.4270 | 0.5684 | 0.2262 | 0.2576 | 0.1719 | 0.9222 | 0.0284 | 0.0249 | 0.0280 | 0.1959 | 0.0279 | 0.1327 |
| Kcnd3 | Kv4.3 | 3.9295 | 5.6806 | 8.2138 | 7.0709 | 2.5401 | 0.4994 | 0.0000 | 0.0006 | 0.0000 | 0.5079 | 0.0319 | 0.0163 |
| Kcne4 | MIRP3 | 2.8336 | 3.3965 | 2.8338 | 3.4998 | 1.5328 | 0.9921 | 0.7588 | 0.7519 | 0.8301 | 0.5306 | 0.9065 | 0.4812 |
| Kcnh2 | ERG-1 | 19.1784 | 23.9876 | 33.2800 | 29.9329 | 13.0732 | 0.9177 | 0.0211 | 0.0127 | 0.0908 | 0.8055 | 0.1065 | 0.3305 |
| Kcnip2 | KChIP2 | 0.8770 | 1.2614 | 6.2452 | 2.4565 | 4.2296 | 0.8503 | 0.0000 | 0.0195 | NA | 0.0000 | NA | 0.0000 |
| Kcnj2 | Kir2.1 | 3.7959 | 5.8069 | 8.9238 | 9.1200 | 10.5562 | 0.5866 | 0.0000 | 0.0062 | 0.0000 | 0.0000 | 0.0004 | 0.0000 |
| Kcnj3 | Kir3.1 | 67.9153 | 106.1186 | 173.6742 | 127.0977 | 14.9386 | 0.5040 | 0.0000 | 0.0000 | 0.0009 | 0.0000 | 0.1607 | 0.0000 |
| Kcnj5 | Kir3.4 | 41.5654 | 62.3068 | 90.9765 | 87.0173 | 10.2758 | 0.5792 | 0.0001 | 0.0000 | 0.0003 | 0.0000 | 0.0000 | 0.0000 |
| Kcnj8 | Kir6.1 | 10.1306 | 12.1079 | 9.4222 | 10.7162 | 14.7107 | 0.9607 | 0.1663 | 0.0573 | 0.7335 | 0.0018 | 0.5134 | 0.0003 |
| Kcnj11 | Kir6.2 | 27.1173 | 33.8028 | 50.6270 | 46.0714 | 40.0770 | 0.9169 | 0.0063 | 0.0000 | 0.0282 | 0.0128 | 0.0000 | 0.0000 |
| Kcnj12 | Kir2.2 | 11.8111 | 16.4495 | 20.8422 | 22.7378 | 7.3871 | 0.6463 | 0.0038 | 0.0005 | 0.0004 | 0.5792 | 0.0000 | 0.0023 |
| Kcnj14 | Kir2.4 | 0.5160 | 0.5936 | 0.2855 | 0.3349 | 1.1657 | 0.9944 | 0.1943 | 0.1767 | 0.1501 | 0.0119 | 0.1260 | 0.0052 |
| Kcnk1 | TWIK-1 | 1.2386 | 1.0025 | 0.1734 | 1.0676 | 0.1514 | 0.6615 | 0.0000 | 0.0001 | 0.6508 | 0.0006 | 0.9005 | 0.0169 |
| Kcnk2 | TREK-1 | 19.1902 | 20.6629 | 31.0402 | 35.2077 | 9.6888 | 0.9490 | 0.0587 | 0.0101 | 0.0079 | 0.1793 | 0.0004 | 0.1262 |
| Kcnk3 | TASK-1 | 81.5361 | 63.5095 | 69.0609 | 50.7278 | 51.5966 | 0.3769 | 0.1341 | 0.6672 | 0.0006 | 0.5159 | 0.1967 | 0.3286 |
| Kcnk5 | TASK-2 | 0.7968 | 1.1251 | 0.4913 | 0.5279 | 0.1827 | 0.7704 | 0.0084 | 0.0002 | 0.0223 | 0.0022 | 0.0006 | 0.0001 |
| Kcnk6 | TWIK2 | 0.1043 | 0.4100 | 0.2699 | 0.0617 | 0.0541 | NA | 0.6761 | 0.8350 | 0.7918 | NA | NA | 0.4548 |
| Kcnn1 | KCNN1 | 1.5978 | 1.2450 | 1.2898 | 1.2316 | 1.7893 | 0.4365 | 0.1376 | 0.9051 | 0.1206 | 0.2821 | 0.9767 | 0.0078 |
| Kcnn2 | KCNN2 | 0.4069 | 0.2746 | 0.3382 | 0.5523 | 0.3523 | 0.7216 | 0.7263 | 0.8267 | 0.7252 | 0.8855 | 0.1430 | 0.3763 |
| Kcnn3 | KCNN3 | 0.8599 | 1.6543 | 0.2393 | 1.4385 | 1.3173 | 0.5402 | 0.0000 | 0.0000 | 0.2019 | 0.1126 | 0.8424 | 0.7527 |
| Kcnn4 | KCNN4 | 2.8904 | 3.4001 | 4.7240 | 3.1767 | 0.5739 | 0.9963 | 0.1374 | 0.3291 | 0.9818 | 0.0003 | 0.9629 | 0.0015 |
| Kcnq1 | Kv7.1 | 31.3850 | 39.5422 | 46.9329 | 38.7546 | 39.3517 | 0.8882 | 0.0746 | 0.1039 | 0.6111 | 0.0358 | 0.9793 | 0.0053 |
| Pln | PLB | 530.3834 | 629.5101 | 680.3749 | 604.6381 | 1517.3616 | 0.9688 | 0.5396 | 0.5675 | 0.9021 | 0.0000 | 0.9675 | 0.0000 |
| Ryr2 | RYR2 | 55.7811 | 93.0507 | 133.0675 | 146.3448 | 83.3721 | 0.1119 | 0.0000 | 0.0002 | 0.0000 | 0.0000 | 0.0000 | 0.0321 |
| Ryr3 | RYR3 | 0.7516 | 1.2036 | 1.1183 | 1.2744 | 0.6819 | 0.2188 | 0.0230 | 0.7533 | 0.0030 | 0.4968 | 0.6926 | 0.4349 |
| Scn1a | NaV1.1 | 0.2914 | 0.5061 | 0.3667 | 0.9816 | 0.8972 | 0.5675 | 0.7654 | 0.3794 | 0.0000 | 0.0000 | 0.0014 | 0.0001 |
| Scn1b | NaVβ1 | 100.7234 | 114.9989 | 104.6469 | 150.4255 | 90.2827 | 0.9926 | 0.8590 | 0.7308 | 0.2437 | 0.6788 | 0.0266 | 0.2174 |
| Scn2a | NaV1.2 | 0.1707 | 0.2675 | 0.1492 | 0.1705 | 0.0961 | 0.6736 | 0.6097 | 0.0603 | 0.8520 | 0.6755 | 0.1556 | 0.1532 |
| Scn2b | NaVβ2 | 0.8997 | 1.9895 | 0.6788 | 1.1850 | 0.4616 | 0.4371 | 0.5159 | 0.0027 | 0.7453 | 0.6645 | 0.1390 | 0.0332 |
| Scn3a | NaV1.3 | 0.3303 | 0.4482 | 0.0848 | 0.2841 | 0.4196 | 0.9408 | 0.0005 | 0.0000 | 0.5459 | 0.4944 | 0.1717 | 0.4722 |
| Scn3b | NaVβ3 | 0.3808 | 0.9157 | 0.2271 | 0.3261 | 0.5835 | 0.2265 | 0.1282 | 0.0000 | 0.5869 | 0.1367 | 0.0007 | 0.9276 |
| Scn4a | NaV1.4 | 0.0846 | 0.1399 | 0.1087 | 0.2205 | 0.1749 | 0.7770 | 0.8250 | 0.7147 | 0.0126 | 0.0440 | 0.2882 | 0.2541 |
| Scn4b | NaVβ4 | 4.4516 | 3.9737 | 1.7143 | 1.4369 | 37.0859 | 0.7337 | 0.0000 | 0.0001 | 0.0000 | 0.0000 | 0.0001 | 0.0000 |
| Scn5a | NaV1.5 | 46.3591 | 67.0760 | 80.9484 | 61.9890 | 48.3884 | 0.5514 | 0.0012 | 0.0919 | 0.3057 | 0.1512 | 0.8144 | 0.6193 |
| Scn7a | NaV2.1 | 12.9837 | 21.1826 | 12.2800 | 7.9008 | 4.7196 | 0.3998 | 0.5398 | 0.0000 | 0.0024 | 0.0102 | 0.0000 | 0.0000 |
| Scn8a | NaV1.6 | 0.0218 | 0.0431 | 0.0168 | 0.0213 | 0.0000 | 0.8779 | 0.8278 | 0.4955 | 0.9138 | NA | 0.4937 | 0.1166 |
| Scn9a | NaV1.7 | 0.0431 | 0.3040 | 0.0188 | 0.0341 | 0.0634 | 0.3259 | 0.4425 | 0.0004 | 0.7933 | 0.6918 | 0.0046 | 0.2124 |
| Scn10a | NaV1.8 | 0.0204 | 0.0846 | 0.0889 | 0.0123 | 0.0000 | 0.3452 | 0.0625 | 0.9577 | 0.6359 | NA | 0.0076 | 0.0120 |
| Scn11a | NaV1.9 | 0.0042 | 0.0024 | 0.0000 | 0.0000 | 0.0000 | NA | NA | NA | NA | NA | NA | NA |

**Cardiac Receptors**

| **Gene Name** | **Protein** | **sSAN** | **iSAN** | **RA** | **LA** | **LV** | **padj (sSAN vs iSAN)** | **padj (sSAN vs RA)** | **padj (iSAN vs RA)** | **padj (sSAN vs LA)** | **padj (sSAN vs LV)** | **padj (iSAN vs LA)** | **padj (iSAN vs LV)** |
| --- | --- | --- | --- | --- | --- | --- | --- | --- | --- | --- | --- | --- | --- |
| Adra1a | ADA1A | 6.6583 | 6.5142 | 8.8541 | 9.9719 | 10.5761 | 0.8290 | 0.5719 | 0.0524 | 0.2302 | 0.0088 | 0.0026 | 0.0000 |
| Adra1b | ADA1A | 10.0827 | 11.2595 | 13.4196 | 16.4799 | 27.3614 | 0.9822 | 0.4548 | 0.1237 | 0.0516 | 0.0000 | 0.0000 | 0.0000 |
| Adra1d | ADA1D | 3.4252 | 2.1572 | 0.6076 | 0.4481 | 5.0826 | 0.5498 | 0.0000 | 0.0098 | 0.0000 | 0.1037 | 0.0000 | 0.0021 |
| Adra2a | ADA2A | 0.3155 | 0.4058 | 0.0319 | 0.1229 | 0.0364 | 0.9511 | 0.0000 | 0.0000 | 0.0388 | 0.0304 | 0.0159 | 0.0110 |
| Adra2b | ADA2B | 0.2965 | 0.2325 | 0.1380 | 0.3146 | 0.4294 | 0.6593 | 0.0329 | 0.4166 | 0.9142 | 0.1216 | 0.4865 | 0.0219 |
| Adra2c | ADA2C | 0.2629 | 0.3536 | 0.0675 | 0.1008 | 0.0936 | 0.9419 | 0.0168 | 0.0240 | 0.1291 | 0.4588 | 0.1323 | 0.3550 |
| Adrb1 | ADRB1 | 16.2290 | 18.6480 | 22.5300 | 21.9175 | 10.2790 | 0.9900 | 0.2068 | 0.2038 | 0.0785 | 0.4288 | 0.0546 | 0.2907 |
| Adrb2 | ADRB2 | 1.8130 | 2.4303 | 1.3148 | 1.9610 | 2.1944 | 0.8690 | 0.0926 | 0.0027 | 0.9520 | 0.2047 | 0.6229 | 0.2601 |
| Adrb3 | ADRB3 | 12.1583 | 5.7706 | 0.0000 | 0.0309 | 0.0000 | 0.6504 | 0.0000 | 0.0147 | 0.0005 | 0.0000 | 0.1000 | 0.0143 |
| Akap13 | AKP13 | 12.2494 | 16.7111 | 19.0544 | 23.8364 | 8.6779 | 0.3785 | 0.0000 | 0.0448 | 0.0000 | 0.6530 | 0.0000 | 0.0332 |
| Ank1 | ANK1 | 21.6813 | 25.5450 | 28.8540 | 29.0878 | 28.2740 | 0.9727 | 0.4514 | 0.2296 | 0.4101 | 0.0533 | 0.1779 | 0.0000 |
| Ank2 | ANK2 | 9.6980 | 14.0879 | 18.3281 | 22.0737 | 11.9120 | 0.3757 | 0.0000 | 0.0011 | 0.0000 | 0.0050 | 0.0000 | 0.0385 |
| Ank3 | ANK3 | 12.2090 | 15.8799 | 22.9793 | 25.0015 | 11.6039 | 0.7811 | 0.0000 | 0.0001 | 0.0000 | 0.2834 | 0.0000 | 0.5778 |
| Atp2b4 | AT2B4 | 10.3225 | 15.7464 | 7.9897 | 8.6725 | 5.0829 | 0.3212 | 0.0000 | 0.0000 | 0.0084 | 0.0068 | 0.0000 | 0.0000 |
| Cav1 | CAV1 | 214.6342 | 205.6570 | 150.6337 | 174.7453 | 148.8934 | 0.5694 | 0.0001 | 0.0003 | 0.0155 | 0.6666 | 0.1898 | 0.5465 |
| Chga | CMGA | 0.1226 | 0.1136 | 0.0067 | 0.0000 | 0.0000 | 0.9344 | 0.0243 | 0.0979 | 0.0120 | 0.1398 | 0.0676 | 0.2108 |
| Chrm1 | ACM1 | 0.0127 | 0.0761 | 0.0000 | 0.0000 | 0.0000 | NA | NA | NA | NA | NA | NA | NA |
| Chrm2 | ACM2 | 45.7027 | 75.5627 | 100.6557 | 113.3595 | 19.9701 | 0.1467 | 0.0000 | 0.0002 | 0.0000 | 0.0056 | 0.0000 | 0.0000 |
| Chrm3 | ACM3 | 0.1534 | 0.1571 | 0.0498 | 0.0652 | 0.0794 | 0.9518 | 0.0345 | 0.1215 | 0.1053 | 0.7344 | 0.2576 | 0.8175 |
| Chrna2 | ACHA2 | 0.1125 | 0.0749 | 0.0329 | 0.0536 | 0.1327 | 0.8393 | 0.2647 | 0.6804 | 0.4019 | 0.7593 | 0.8641 | 0.4585 |
| Chrna4 | ACHA4 | 0.0286 | 0.0330 | 0.0082 | 0.0385 | 0.0397 | NA | NA | NA | 0.8948 | NA | 0.9352 | 0.7707 |
| Chrna7 | ACHA7 | 0.0627 | 0.9050 | 0.0993 | 0.1407 | 0.0813 | 0.0023 | 0.8722 | 0.0060 | 0.6149 | 0.7766 | 0.0076 | 0.0055 |
| Chrnb2 | ACHB2 | 0.1031 | 0.0991 | 0.0345 | 0.0412 | 0.0247 | 0.9693 | 0.4178 | 0.5368 | 0.3922 | 0.5477 | 0.5512 | 0.5820 |
| Chrnb4 | ACHB4 | 1.1325 | 0.6506 | 0.0000 | 0.0099 | 0.0000 | 0.8834 | 0.0000 | 0.4561 | 0.0000 | 0.0000 | 0.1755 | 0.4956 |
| Chrne | ACHE | 0.0199 | 0.0841 | 0.0281 | 0.0835 | 0.0931 | NA | NA | 0.4785 | 0.3232 | NA | 0.9891 | 0.7061 |
| Gnai2 | GNAI2 | 141.2814 | 158.7870 | 164.8379 | 167.8917 | 137.8831 | 0.9419 | 0.7526 | 0.4682 | 0.5623 | 0.1229 | 0.1259 | 0.0006 |
| Myh6 | MHC-α | 1775.1071 | 2615.5120 | 3933.1945 | 3786.7204 | 2916.5756 | 0.5909 | 0.0000 | 0.0001 | 0.0000 | 0.0004 | 0.0006 | 0.0003 |
| Myl1 | MLC1 | 10.5530 | 26.0630 | 9.4177 | 8.6830 | 1.2086 | 0.2232 | 0.6831 | 0.0694 | 0.4066 | 0.0000 | 0.0000 | 0.0000 |
| Myl2 | MLC2 | 10.8466 | 8.6661 | 0.3977 | 0.0984 | 3779.3539 | 0.8535 | 0.0000 | 0.0000 | 0.0000 | 0.0000 | 0.0000 | 0.0000 |
| Pde4b | PDE4B | 8.4845 | 11.4462 | 16.6925 | 15.5820 | 6.9080 | 0.7301 | 0.0000 | 0.0044 | 0.0000 | 0.8048 | 0.0114 | 0.6836 |
| Pde4d | PDE4D | 15.7715 | 11.8259 | 11.9324 | 8.8635 | 7.1702 | 0.7812 | 0.4885 | 0.9776 | 0.1939 | 0.5579 | 0.2598 | 0.7601 |
| Ramp3 | RAMP3 | 1.4795 | 3.2487 | 0.8384 | 0.8480 | 0.3874 | 0.2744 | 0.0503 | 0.0000 | 0.0790 | 0.0652 | 0.0000 | 0.0001 |
| Rgs2 | RGS2 | 27.8817 | 25.3996 | 27.8203 | 31.7811 | 4.8809 | 0.8204 | 0.6983 | 0.8197 | 0.9669 | 0.0000 | 0.4343 | 0.0000 |
| Rgs6 | RGS6 | 29.7601 | 42.7836 | 66.5547 | 42.8896 | 10.8748 | 0.7276 | 0.0004 | 0.0003 | 0.2579 | 0.0146 | 0.9100 | 0.0000 |
| Src | SRC | 6.6441 | 8.9090 | 6.2423 | 7.2484 | 3.8464 | 0.6232 | 0.2408 | 0.0088 | 0.9137 | 0.1846 | 0.1581 | 0.0079 |
| Tnnc1 | TN-C | 1262.1144 | 1549.9985 | 1964.4461 | 1829.1876 | 2412.7265 | 0.9426 | 0.1798 | 0.0048 | 0.2967 | 0.0020 | 0.0343 | 0.0000 |
| Tpm1 | TPM1 | 2571.0690 | 3212.4937 | 4012.9787 | 3435.6641 | 4818.8587 | 0.8954 | 0.0385 | 0.0144 | 0.3008 | 0.0000 | 0.4437 | 0.0000 |
| Vip | VIP | 0.1188 | 0.0988 | 0.0761 | 0.1065 | 0.0525 | NA | 0.6182 | 0.8810 | 0.8519 | 0.7620 | 0.9578 | 0.9083 |

**Neuronal Proteins**

| **Gene Name** | **Protein** | **sSAN** | **iSAN** | **RA** | **LA** | **LV** | **padj (sSAN vs iSAN)** | **padj (sSAN vs RA)** | **padj (iSAN vs RA)** | **padj (sSAN vs LA)** | **padj (sSAN vs LV)** | **padj (iSAN vs LA)** | **padj (iSAN vs LV)** |
| --- | --- | --- | --- | --- | --- | --- | --- | --- | --- | --- | --- | --- | --- |
| Cadps | CAPS1 | 0.5657 | 1.3120 | 1.0657 | 0.6607 | 2.1193 | 0.0497 | 0.0242 | 0.5116 | 0.9077 | 0.0000 | 0.0050 | 0.0003 |
| Chat | CHAT | 0.0055 | 0.2050 | 0.0000 | 0.0000 | 0.0000 | 0.4505 | NA | 0.1154 | NA | NA | 0.1050 | 0.1826 |
| Ctnnb1 | CTNNB1 | 84.1409 | 88.9037 | 98.7676 | 101.7157 | 77.6451 | 0.7083 | 0.7641 | 0.0949 | 0.5280 | 0.2548 | 0.0317 | 0.0040 |
| Dbh | DOPO | 0.5524 | 1.2755 | 0.5229 | 0.4740 | 0.5617 | 0.6504 | 0.6999 | 0.1735 | 0.5278 | 0.6826 | 0.1216 | 0.6067 |
| Ddc | DDC | 13.8608 | 17.0017 | 12.1921 | 7.3774 | 7.6967 | 0.9452 | 0.4026 | 0.0007 | 0.0007 | 0.4412 | 0.0000 | 0.0146 |
| Erbb2 | ERBB2 | 1.9499 | 2.6758 | 3.8769 | 4.0866 | 0.8842 | 0.7321 | 0.0006 | 0.0002 | 0.0001 | 0.1113 | 0.0000 | 0.0002 |
| Fzd3 | FZD3 | 0.8150 | 1.2139 | 1.3975 | 1.9392 | 0.6214 | 0.7841 | 0.1899 | 0.5032 | 0.0046 | 0.9831 | 0.0020 | 0.4656 |
| Gata2 | GATA2 | 4.8870 | 5.3822 | 5.2309 | 5.2149 | 4.0165 | 0.9371 | 0.8034 | 0.9345 | 0.8315 | 0.8135 | 0.9709 | 0.4889 |
| Gata3 | GATA3 | 0.4330 | 0.6050 | 0.5747 | 0.2643 | 0.4931 | 0.8998 | 0.7684 | 0.9708 | 0.0959 | 0.4846 | 0.0468 | 0.7303 |
| Gbx2 | GBX2 | 2.5987 | 0.6311 | 0.0604 | 0.0115 | 0.0730 | 0.5913 | 0.0000 | NA | 0.0000 | 0.0002 | NA | 0.2793 |
| Gdnf | GDNF | 0.0343 | 0.0901 | 0.0749 | 0.0329 | 0.0000 | NA | NA | NA | NA | NA | NA | NA |
| Gfra2 | GFRA2 | 3.6275 | 4.6978 | 2.2616 | 2.5373 | 2.2239 | 0.8994 | 0.0011 | 0.0001 | 0.0224 | 0.5196 | 0.0025 | 0.2174 |
| Gfra3 | GFRA3 | 3.1340 | 5.0434 | 2.0318 | 1.8120 | 1.6127 | 0.5457 | 0.0105 | 0.0000 | 0.0045 | 0.3297 | 0.0000 | 0.0094 |
| Hand2 | HAND2 | 14.3168 | 20.0224 | 24.0825 | 26.1342 | 21.3698 | 0.7720 | 0.0801 | 0.1135 | 0.0308 | 0.0371 | 0.0200 | 0.0025 |
| Nav2 | NAV2 | 3.1797 | 3.9903 | 7.7448 | 8.5294 | 2.8925 | 0.9313 | 0.0000 | 0.0002 | 0.0000 | 0.5466 | 0.0000 | 0.7514 |
| Nefm | NFM | 0.1049 | 1.4269 | 0.0041 | 0.0115 | 0.0000 | 0.4371 | 0.0129 | 0.1353 | 0.0316 | 0.0931 | 0.1005 | 0.2885 |
| Nf1 | NF1 | 3.4166 | 4.4226 | 5.0893 | 5.0143 | 2.2369 | 0.8335 | 0.0809 | 0.1034 | 0.1094 | 0.5498 | 0.1768 | 0.0417 |
| Ngf | NGF | 2.1233 | 2.3106 | 2.1447 | 1.5297 | 1.6650 | 0.9605 | 0.7969 | 0.8756 | 0.1761 | 0.9708 | 0.1303 | 0.8278 |
| Nos1 | NOS1 | 1.0169 | 2.5999 | 0.9026 | 0.2618 | 0.1701 | 0.3191 | 0.6765 | 0.0403 | 0.0041 | 0.0030 | 0.0000 | 0.0000 |
| Nppa | NPPA | 13411.9419 | 19244.9247 | 40980.0987 | 31805.8394 | 343.9898 | 0.8536 | 0.0022 | 0.0000 | 0.0268 | 0.0000 | 0.0004 | 0.0000 |
| Nppb | NPPB | 261.8293 | 351.3728 | 1025.9621 | 587.7179 | 366.7850 | 0.9475 | 0.0180 | 0.0015 | 0.2681 | 0.5038 | 0.2805 | 0.3286 |
| Npy | NPY | 0.0602 | 1.2748 | 0.1093 | 0.0402 | 0.0502 | NA | NA | NA | NA | NA | NA | NA |
| Nrp1 | NRP1 | 44.4426 | 41.5970 | 42.7745 | 46.8042 | 53.4783 | 0.4873 | 0.2746 | 0.7145 | 0.6950 | 0.0258 | 0.0413 | 0.0000 |
| Nrp2 | NRP2 | 8.9378 | 7.5372 | 5.1420 | 6.4375 | 8.3950 | 0.5553 | 0.0022 | 0.0479 | 0.0488 | 0.6846 | 0.5524 | 0.0139 |
| Ntm | NTM | 0.3494 | 0.3285 | 0.0789 | 0.0822 | 0.1299 | 0.8598 | 0.0001 | 0.0004 | 0.0001 | 0.2930 | 0.0005 | 0.4245 |
| Ntrk1 | NTRK1 | 0.1234 | 0.3648 | 0.0150 | 0.0131 | 0.0000 | 0.6711 | NA | 0.0315 | NA | NA | 0.0260 | 0.1098 |
| Phox2a | PHOX2A | 0.4650 | 0.8920 | 0.3732 | 0.3604 | 0.5223 | 0.7957 | 0.6689 | 0.2284 | 0.6111 | 0.6756 | 0.1880 | 0.8824 |
| Phox2b | PHOX2B | 0.1652 | 0.5085 | 0.0342 | 0.0000 | 0.0000 | 0.7268 | 0.3383 | 0.0536 | 0.2184 | NA | 0.0360 | 0.1195 |
| Plxna4 | PLXNA4 | 2.8573 | 4.5128 | 5.8000 | 4.6645 | 0.9968 | 0.4531 | 0.0006 | 0.0370 | 0.0734 | 0.0026 | 0.8171 | 0.0000 |
| Sema3a | SEMA3A | 2.4851 | 4.1040 | 3.7278 | 5.7508 | 0.7311 | 0.1781 | 0.0645 | 0.7241 | 0.0000 | 0.0001 | 0.0014 | 0.0000 |
| Sema3c | SEMA3C | 20.6402 | 34.1153 | 13.4876 | 19.7140 | 12.3460 | 0.3846 | 0.0001 | 0.0000 | 0.3551 | 0.3021 | 0.0002 | 0.0010 |
| Sema3f | SEMA3F | 5.5364 | 7.9129 | 6.1002 | 5.4480 | 2.3384 | 0.4731 | 0.8849 | 0.0842 | 0.4894 | 0.0000 | 0.0379 | 0.0000 |
| Sox10 | SOX10 | 2.5874 | 2.6104 | 1.0332 | 1.3508 | 0.9846 | 0.8780 | 0.0000 | 0.0002 | 0.0005 | 0.0167 | 0.0085 | 0.0578 |
| Sox11 | SOX11 | 0.0591 | 0.1251 | 0.1365 | 0.2920 | 0.0253 | 0.8494 | 0.6302 | 0.9649 | 0.0768 | NA | 0.2877 | 0.4661 |
| Tbx1 | TBX1 | 0.2098 | 0.2185 | 0.0819 | 0.0748 | 0.1327 | 0.9823 | 0.5133 | 0.3883 | 0.2977 | 0.9313 | 0.1640 | 0.9319 |
| Tfap2a | AP2A | 0.1276 | 0.1685 | 0.1095 | 0.0733 | 0.0077 | 0.9600 | 0.7742 | 0.6372 | 0.4012 | 0.1006 | 0.2910 | 0.0583 |
| Tfap2b | AP2B | 0.0896 | 0.1397 | 0.0598 | 0.0480 | 0.0232 | 0.8780 | 0.4186 | 0.2127 | 0.2383 | 0.3170 | 0.1052 | 0.1562 |
| Th | TH | 0.5223 | 0.1954 | 0.0000 | 0.0000 | 0.0144 | 0.3083 | 0.0000 | 0.0967 | 0.0000 | 0.0006 | 0.0874 | 0.2809 |
| Tp63 | P63 | 0.0331 | 0.0457 | 0.0645 | 0.0736 | 0.0000 | NA | 0.6395 | 0.7693 | 0.5441 | NA | 0.6658 | NA |
| Uchl1 | UCHL1 | 2.5198 | 6.8831 | 0.9337 | 0.8533 | 0.2313 | 0.4504 | 0.0014 | 0.0131 | 0.0000 | 0.0000 | 0.0029 | 0.0062 |
| Vcam1 | VCAM1 | 10.3056 | 13.0153 | 15.9071 | 13.3704 | 2.1373 | 0.8967 | 0.0021 | 0.1766 | 0.2153 | 0.0000 | 0.8043 | 0.0000 |
| Vsnl1 | VISL1 | 28.4080 | 28.3516 | 31.0360 | 23.7078 | 1.2023 | 0.8501 | 0.9660 | 0.4804 | 0.2068 | 0.0000 | 0.3545 | 0.0000 |

**Transcription Factors**

| **Gene Name** | **Protein** | **sSAN** | **iSAN** | **RA** | **LA** | **LV** | **padj (sSAN vs iSAN)** | **padj (sSAN vs RA)** | **padj (iSAN vs RA)** | **padj (sSAN vs LA)** | **padj (sSAN vs LV)** | **padj (iSAN vs LA)** | **padj (iSAN vs LV)** |
| --- | --- | --- | --- | --- | --- | --- | --- | --- | --- | --- | --- | --- | --- |
| Bmp1 | BMP1 | 12.8471 | 21.1851 | 12.8695 | 14.3738 | 5.4179 | 0.0147 | 0.4638 | 0.0000 | 0.9750 | 0.0005 | 0.0001 | 0.0000 |
| Bmp2 | BMP2 | 1.9524 | 2.7283 | 2.3386 | 2.0820 | 0.4778 | 0.8113 | 0.8084 | 0.5965 | 0.9322 | 0.0012 | 0.2842 | 0.0000 |
| Bmp4 | BMP4 | 10.6098 | 16.6180 | 7.7217 | 8.7917 | 4.1805 | 0.6093 | 0.0386 | 0.0000 | 0.1652 | 0.0227 | 0.0005 | 0.0001 |
| Cpne5 | CPNE5 | 1.6459 | 0.7918 | 0.1392 | 0.1403 | 0.0697 | 0.1829 | 0.0000 | 0.0000 | 0.0000 | 0.0000 | 0.0000 | 0.0000 |
| Csrp2 | CRP2 | 9.0276 | 10.3498 | 3.7935 | 5.1309 | 5.0726 | 0.9935 | 0.0001 | 0.0000 | 0.0120 | 0.5028 | 0.0000 | 0.1952 |
| Dll1 | DLL1 | 1.3639 | 0.9658 | 0.3771 | 0.5329 | 1.2150 | 0.3259 | 0.0000 | 0.0000 | 0.0000 | 0.8028 | 0.0089 | 0.0084 |
| Fgfr1 | FGFR1 | 47.8929 | 52.1090 | 35.1096 | 38.6117 | 20.6246 | 0.9175 | 0.0023 | 0.0001 | 0.0247 | 0.0039 | 0.0041 | 0.0001 |
| Igfbp5 | IBP5 | 375.5620 | 597.0261 | 537.2675 | 307.7379 | 70.2938 | 0.1061 | 0.0052 | 0.5392 | 0.0083 | 0.0000 | 0.0000 | 0.0000 |
| Isl1 | ISL1 | 0.3940 | 0.8870 | 0.0051 | 0.0000 | 0.0000 | 0.5911 | 0.0000 | 0.0000 | 0.0000 | 0.0001 | 0.0000 | 0.0000 |
| Lbh | LBH | 65.0875 | 71.1720 | 87.9223 | 113.6710 | 513.3480 | 0.9511 | 0.4165 | 0.1055 | 0.0155 | 0.0000 | 0.0000 | 0.0000 |
| Nkx2-5 | NKX25 | 43.7881 | 50.1414 | 81.0965 | 72.8121 | 50.2877 | 0.9915 | 0.0580 | 0.0000 | 0.1540 | 0.3111 | 0.0000 | 0.0004 |
| Nodal | NODAL | 0.0000 | 0.0068 | 0.0066 | 0.0000 | 0.0000 | NA | NA | NA | NA | NA | NA | NA |
| Notch3 | NOTC3 | 7.4479 | 7.7192 | 4.3242 | 6.2374 | 7.6032 | 0.7640 | 0.0000 | 0.0000 | 0.0112 | 0.3116 | 0.1500 | 0.0565 |
| Pitx2 | PITX2 | 1.9191 | 2.3888 | 0.0555 | 12.3048 | 0.2500 | 0.9591 | 0.0000 | 0.0000 | 0.0000 | 0.0000 | 0.0000 | 0.0000 |
| Rec114 | REC114 | 8.3433 | 9.1578 | 3.2576 | 0.9617 | 3.0539 | 0.9822 | 0.0007 | 0.0000 | 0.0000 | 0.1901 | 0.0000 | 0.0358 |
| Shox2 | SHOX2 | 25.2456 | 32.3653 | 1.7902 | 1.0545 | 0.0907 | 0.8636 | 0.0000 | 0.0000 | 0.0000 | 0.0000 | 0.0000 | 0.0000 |
| Slc9a3r2 | NHRF2 | 45.0323 | 42.4191 | 31.7430 | 37.6515 | 54.2023 | 0.5440 | 0.0001 | 0.0014 | 0.0293 | 0.0253 | 0.4426 | 0.0000 |
| Smad1 | SMAD1 | 7.4446 | 8.5958 | 8.1787 | 9.3718 | 8.2892 | 0.9915 | 0.8662 | 0.8248 | 0.4010 | 0.0482 | 0.3873 | 0.0105 |
| Smad6 | SMAD6 | 20.6431 | 21.3248 | 34.7682 | 12.0542 | 5.7974 | 0.9245 | 0.0997 | 0.0000 | 0.0220 | 0.0027 | 0.0014 | 0.0000 |
| Smad9 | SMAD9 | 0.1430 | 0.3340 | 0.5949 | 0.1307 | 0.0768 | 0.7743 | 0.0233 | 0.4705 | 0.9060 | 0.8549 | 0.3883 | 0.4084 |
| Smoc2 | SMOC2 | 9.7980 | 8.2379 | 1.6050 | 7.5885 | 5.3434 | 0.2889 | 0.0000 | 0.0000 | 0.0119 | 0.2831 | 0.7411 | 0.9175 |
| Sox8 | SOX8 | 0.2852 | 0.4705 | 0.3720 | 0.1884 | 0.1899 | 0.6702 | 0.7453 | 0.6490 | 0.2158 | 0.8506 | 0.0230 | 0.3648 |
| Tbx3 | TBX3 | 6.7225 | 6.2828 | 1.5959 | 1.2873 | 2.6189 | 0.6776 | 0.0000 | 0.0000 | 0.0000 | 0.0039 | 0.0000 | 0.0330 |
| Tbx5 | TBX5 | 35.1108 | 47.3619 | 73.0955 | 58.3263 | 6.4525 | 0.7605 | 0.0000 | 0.0000 | 0.0085 | 0.0000 | 0.0325 | 0.0000 |
| Tbx18 | TBX18 | 4.0979 | 5.5194 | 5.2842 | 4.0586 | 0.9822 | 0.8428 | 0.5148 | 0.9623 | 0.5881 | 0.0000 | 0.1691 | 0.0000 |
| Tbx20 | TBX20 | 32.1895 | 63.9812 | 93.2214 | 57.9561 | 18.3026 | 0.0244 | 0.0000 | 0.0003 | 0.0023 | 0.3343 | 0.6836 | 0.0000 |
| Tenm4 | TN4 | 0.3007 | 0.3793 | 0.1318 | 0.3185 | 0.0228 | 0.9568 | 0.0158 | 0.0004 | 0.9280 | 0.0001 | 0.7566 | 0.0000 |
| Vwf | VWF | 33.9929 | 58.6170 | 51.6029 | 42.3021 | 8.5395 | 0.2211 | 0.0158 | 0.6981 | 0.7031 | 0.0000 | 0.4245 | 0.0000 |
| Wnt1 | WNT1 | 0.0364 | 0.0446 | 0.0053 | 0.0098 | 0.0000 | NA | NA | NA | NA | NA | NA | NA |

**Supplementary Table 3:** Comparisons of weights, dimensions, and thicknesses between normal and failing male rat hearts.

| **Measurement** | **Normal (n = 7)** | **Failing (n = 6)** | **p value** |
| --- | --- | --- | --- |
| Body weight (g) | 413.4 ± 49.8 | 362.8 ± 29.67 | ns |
| Heart weight (g) | 1.87 ± 0.24 | 3.36 ± 0.21 | 0.0001 |
| Heart/body weight (%) | 0.45 ± 0.02 | 0.95 ±0.08 | 0.0001 |
| **Measurement (mm)** | **Normal (n = 12)** | **Failing (n = 10)** | **p value** |
| Heart width | 14.29 ± 0.59 | 16.23 ± 1.27 | 0.01 |
| Hearth length | 22.18 ± 1.171 | 25.01 ± 2.25 | 0.002 |
| Septum thickness | 3.93 ± 0.66 | 4.81 ± 0.55 | 0.006 |
| LV wall thickness | 4.29 ± 0.84 | 5.92 ± 0.74 | <0.0001 |
| RV wall thickness | 1.71 ± 0.46 | 1.88 ± 0.36 | ns |

**Supplementary Table 4:** Average FPKM values of the cardiac genes of interest for the human heart and associated padj values between groups.

| Gene Name | Protein | sSAN | iSAN | RA | LA | padj sSAN vs iSAN | padj sSAN vs RA | padj sSAN vs LA | padj iSAN vs RA | padj iSAN vs LA | padj RA vs LA |
| --- | --- | --- | --- | --- | --- | --- | --- | --- | --- | --- | --- |
| ATP2A2 | SERCA2A | 4.9E-02 | 5.5E-02 | 4.8E-02 | 5.0E-02 | 0.9998 | 0.3919 | 0.1310 | 0.7505 | 0.4048 | 0.9999 |
| CACNA1A | Cav2.1 | 3.7E-06 | 2.6E-06 | 2.9E-06 | 1.7E-06 | 0.9998 | 0.8877 | 0.6646 | 0.5369 | 0.9856 | 0.9999 |
| CACNA1C | Cav1.2 | 1.5E-04 | 1.7E-04 | 1.2E-04 | 1.0E-04 | 0.9998 | 0.8275 | 0.8855 | 0.9864 | 0.9210 | 0.9999 |
| CACNA1D | Cav1.3 | 1.3E-05 | 1.4E-05 | 6.3E-06 | 3.7E-06 | 0.9998 | 0.6538 | 0.2368 | 0.5291 | 0.1276 | 0.9999 |
| CACNA1G | Cav3.1 | 1.4E-04 | 9.8E-05 | 1.0E-04 | 1.7E-04 | 0.9998 | 0.9890 | 0.4206 | 0.6821 | 0.1334 | 0.9999 |
| CACNA1H | Cav3.2 | 3.8E-04 | 3.6E-04 | 3.2E-04 | 4.4E-04 | 0.9998 | 0.7200 | 0.0505 | 0.7415 | 0.0434 | 0.9999 |
| CACNA2D1 | Cavα2δ1 | 2.3E-04 | 2.2E-04 | 1.1E-04 | 9.1E-05 | 0.9998 | 0.2109 | 0.0918 | 0.1512 | 0.0381 | 0.9999 |
| CACNA2D2 | Cavα2δ2 | 2.3E-04 | 3.0E-04 | 1.2E-04 | 5.2E-05 | 0.9998 | 0.5790 | 0.0327 | 0.2303 | 0.0046 | 0.7206 |
| CACNA2D3 | Cavα2δ3 | 3.9E-05 | 4.5E-05 | 4.8E-05 | 3.3E-05 | 0.9998 | 0.0381 | 0.5185 | 0.2005 | 0.8488 | 0.9999 |
| CACNB1 | Cavβ1 | 2.4E-04 | 2.2E-04 | 1.1E-04 | 1.3E-04 | 0.9998 | 0.3919 | 0.8905 | 0.3420 | 0.9103 | 0.9999 |
| CACNB2 | Cavβ2 | 7.1E-05 | 9.1E-05 | 8.1E-05 | 5.8E-05 | 0.9998 | 0.2751 | 0.6833 | 0.6847 | 0.9576 | 0.9999 |
| CACNB3 | Cavβ3 | 3.3E-04 | 2.4E-04 | 1.9E-04 | 2.3E-04 | 0.9998 | 0.7762 | 0.8280 | 0.9286 | 0.3738 | 0.9999 |
| CACNG4 | Cavγ4 | 1.6E-06 | 6.9E-06 | 2.9E-06 | 1.2E-06 | 0.9998 | NA | NA | 0.8462 | 0.5997 | NA |
| CACNG7 | Cavγ7 | 6.1E-06 | 2.6E-06 | 3.7E-07 | 2.9E-07 | 0.9998 | NA | NA | NA | NA | NA |
| CALM1 | Calm1 | 4.7E-02 | 4.5E-02 | 3.4E-02 | 3.6E-02 | 0.9998 | 0.9257 | 0.3541 | 0.9650 | 0.4055 | 0.9999 |
| CALM3 | Calm3 | 2.4E-02 | 2.7E-02 | 2.5E-02 | 2.1E-02 | 0.9998 | 0.2180 | 0.2285 | 0.5738 | 0.5544 | 0.9999 |
| CASQ2 | Casq2 | 1.4E-02 | 1.8E-02 | 1.4E-02 | 1.4E-02 | 0.9998 | 0.1718 | 0.1149 | 0.8868 | 0.7592 | 0.9999 |
| GJA1 | Cx43 | 2.1E-02 | 2.2E-02 | 1.4E-02 | 1.5E-02 | 0.9998 | 0.9011 | 0.8445 | 0.8270 | 0.9980 | 0.9999 |
| GJA5 | Cx40 | 1.3E-03 | 1.5E-03 | 1.6E-03 | 1.7E-03 | 0.9998 | 0.3757 | 0.2651 | 0.4561 | 0.3395 | 0.9999 |
| GJD3 | Cx30.2 | 2.8E-04 | 3.0E-04 | 3.0E-04 | 3.0E-04 | 0.9998 | 0.5027 | 0.3861 | 0.7235 | 0.5612 | 0.9999 |
| HCN1 | HCN1 | 4.0E-05 | 3.1E-05 | 3.3E-05 | 2.3E-05 | 0.9998 | 0.7492 | 0.8022 | 0.1794 | 0.8639 | 0.9999 |
| HCN2 | HCN2 | 6.4E-04 | 7.2E-04 | 8.7E-04 | 2.5E-04 | 0.9998 | 0.3601 | 0.2883 | 0.5413 | 0.0705 | 0.0693 |
| HCN3 | HCN3 | 1.2E-04 | 6.4E-05 | 5.1E-05 | 5.4E-05 | 0.9998 | 0.5442 | 0.5921 | 0.9570 | 0.7656 | 0.9999 |
| HCN4 | HCN4 | 9.3E-04 | 1.0E-03 | 7.6E-04 | 3.0E-04 | 0.9998 | 0.8864 | 0.2088 | 0.9607 | 0.1841 | 0.7231 |
| ITPR1 | IP3R1 | 1.6E-04 | 1.3E-04 | 8.1E-05 | 6.9E-05 | 0.9998 | 0.3678 | 0.1241 | 0.8109 | 0.5214 | 0.9999 |
| ITPR2 | IP3R2 | 9.0E-05 | 7.5E-05 | 3.9E-05 | 3.1E-05 | 0.9998 | 0.0746 | 0.0628 | 0.2393 | 0.1760 | 0.9999 |
| ITPR3 | IP3R3 | 2.6E-04 | 2.4E-04 | 2.0E-04 | 2.2E-04 | 0.9998 | 0.9081 | 0.3859 | 0.8861 | 0.3703 | 0.9999 |
| KCNA2 | Kv1.2 | 3.2E-05 | 1.6E-05 | 3.0E-05 | 2.5E-05 | 0.9998 | 0.7262 | 0.8158 | 0.1561 | 0.2886 | NA |
| KCNA3 | Kv1.3 | 2.0E-04 | 2.4E-04 | 2.4E-04 | 3.5E-04 | 0.9998 | 0.6780 | 0.3065 | 0.8383 | 0.4652 | 0.9999 |
| KCNA4 | Kv1.4 | 1.1E-05 | 7.4E-06 | 2.0E-04 | 1.3E-04 | 0.9998 | 0.0000 | 0.0002 | 0.0001 | 0.0005 | 0.9999 |
| KCNA5 | Kv1.5 | 1.7E-02 | 2.2E-02 | 2.8E-02 | 2.4E-02 | 0.9998 | 0.0001 | 0.0009 | 0.0333 | 0.0681 | 0.9999 |
| KCNB1 | Kv2.1 | 3.1E-05 | 3.2E-05 | 2.0E-05 | 2.6E-05 | 0.9998 | 0.9671 | 0.6743 | 0.9366 | 0.7965 | 0.9999 |
| KCND1 | Kv4.1 | 7.5E-04 | 5.7E-04 | 2.9E-04 | 1.4E-04 | 0.9998 | 0.3182 | 0.0243 | 0.6482 | 0.0980 | 0.9999 |
| KCND3 | Kv4.3 | 2.1E-04 | 2.3E-04 | 2.2E-04 | 1.9E-04 | 0.9998 | 0.2270 | 0.2781 | 0.5338 | 0.5612 | 0.9999 |
| KCNE4 | MIRP3 | 1.7E-04 | 1.3E-04 | 3.1E-05 | 3.2E-05 | 0.9998 | 0.0007 | 0.0020 | 0.0013 | 0.0068 | 0.9999 |
| KCNH2 | ERG-1 | 4.2E-03 | 5.1E-03 | 4.6E-03 | 3.4E-03 | 0.9998 | 0.0122 | 0.3903 | 0.5561 | 0.9972 | 0.9999 |
| KCNIP2 | KChIP2 | 1.2E-02 | 1.4E-02 | 2.4E-02 | 2.1E-02 | 0.9998 | 0.0217 | 0.0376 | 0.0241 | 0.0657 | 0.9999 |
| KCNJ2 | Kir2.1 | 4.5E-04 | 3.9E-04 | 5.6E-04 | 4.3E-04 | 0.9998 | 0.1046 | 0.4390 | 0.2191 | 0.4459 | 0.9999 |
| KCNJ3 | Kir3.1 | 6.8E-04 | 8.3E-04 | 5.4E-04 | 4.7E-04 | 0.9998 | 0.8200 | 0.8336 | 0.8540 | 0.6961 | 0.9999 |
| KCNJ5 | Kir3.4 | 9.3E-04 | 1.2E-03 | 9.8E-04 | 5.5E-04 | 0.9998 | 0.1332 | 0.8777 | 0.8538 | 0.4648 | 0.7924 |
| KCNJ8 | Kir6.1 | 2.1E-03 | 2.2E-03 | 2.9E-03 | 2.0E-03 | 0.9998 | 0.1348 | 0.3359 | 0.2576 | 0.5161 | 0.9999 |
| KCNJ11 | Kir6.2 | 5.0E-03 | 4.8E-03 | 5.4E-03 | 5.4E-03 | 0.9998 | 0.2143 | 0.0399 | 0.2237 | 0.0207 | 0.9999 |
| KCNJ12 | Kir2.2 | 3.5E-04 | 3.6E-04 | 4.7E-04 | 4.5E-04 | 0.9998 | 0.0460 | 0.0095 | 0.0566 | 0.0082 | 0.9999 |
| KCNJ14 | Kir2.4 | 6.1E-05 | 3.8E-05 | 1.2E-05 | 2.1E-05 | 0.9998 | 0.2745 | 0.5635 | 0.6353 | 0.8889 | NA |
| KCNK1 | TWIK-1 | 1.2E-03 | 1.4E-03 | 1.3E-03 | 1.1E-03 | 0.9998 | 0.1369 | 0.1861 | 0.4534 | 0.5036 | 0.9999 |
| KCNK2 | TREK-1 | 5.8E-06 | 2.2E-06 | 5.0E-07 | 1.6E-06 | 0.9998 | 0.0424 | 0.4223 | 0.5334 | 0.9831 | 0.9999 |
| KCNK3 | TASK-1 | 1.1E-03 | 1.0E-03 | 9.7E-04 | 1.4E-03 | 0.9998 | 0.6756 | 0.1158 | 0.6675 | 0.0875 | 0.9999 |
| KCNK5 | TASK-2 | 1.1E-04 | 1.0E-04 | 4.8E-05 | 2.8E-05 | 0.9998 | 0.4654 | 0.0851 | 0.5432 | 0.1113 | 0.9999 |
| KCNK6 | TWIK2 | 8.0E-04 | 5.0E-04 | 4.5E-04 | 3.9E-04 | 0.9998 | 0.7783 | 0.7547 | 0.7763 | 0.8017 | 0.9999 |
| KCNN1 | KCNN1 | 3.1E-05 | 2.6E-05 | 3.5E-05 | 3.8E-05 | 0.9998 | 0.5884 | 0.2511 | 0.4565 | 0.0979 | 0.9999 |
| KCNN2 | KCNN2 | 3.8E-05 | 4.1E-05 | 4.7E-05 | 6.3E-05 | 0.9998 | 0.0830 | 0.0002 | 0.1744 | 0.0003 | 0.9478 |
| KCNN3 | KCNN3 | 4.6E-05 | 3.5E-05 | 1.9E-05 | 2.3E-05 | 0.9998 | 0.3498 | 0.7150 | 0.6780 | 0.9691 | 0.9999 |
| KCNN4 | KCNN4 | 1.8E-04 | 1.7E-04 | 2.2E-05 | 3.7E-05 | 0.9998 | 0.0138 | 0.1497 | NA | 0.2220 | 0.9999 |
| KCNQ1 | Kv7.1 | 1.1E-04 | 1.4E-04 | 1.1E-04 | 9.1E-05 | 0.9998 | 0.1047 | 0.3576 | 0.8391 | 0.9959 | 0.9999 |
| PLN | PLB | 1.2E-01 | 1.4E-01 | 1.0E-01 | 1.0E-01 | 0.9998 | 0.7643 | 0.5059 | 0.9639 | 0.8931 | 0.9999 |
| RYR2 | RYR2 | 1.9E-03 | 2.2E-03 | 1.8E-03 | 1.5E-03 | 0.9998 | 0.5568 | 0.7126 | 0.9406 | 0.9536 | 0.9999 |
| RYR3 | RYR3 | 4.7E-06 | 1.8E-06 | 1.2E-06 | 1.4E-06 | 0.9998 | 0.0535 | 0.1038 | 0.9437 | 0.8891 | 0.9999 |
| SCN1A | NaV1.1 | 1.7E-06 | 1.7E-06 | 2.5E-06 | 9.7E-07 | 0.9998 | 0.5672 | 0.9458 | 0.6482 | 0.9380 | 0.9999 |
| SCN1B | NaVβ1 | 4.4E-03 | 4.8E-03 | 1.0E-02 | 7.5E-03 | 0.9998 | 0.0000 | 0.0001 | 0.0009 | 0.0047 | 0.9999 |
| SCN2A | NaV1.2 | 5.7E-06 | 1.7E-06 | 9.6E-07 | 6.4E-07 | 0.9998 | 0.1313 | 0.1460 | 0.9203 | 0.8116 | NA |
| SCN2B | NaVβ2 | 1.2E-03 | 1.6E-03 | 7.4E-04 | 5.5E-04 | 0.9998 | 0.9385 | 0.7136 | 0.5022 | 0.3448 | 0.9999 |
| SCN3A | NaV1.3 | 6.4E-05 | 5.2E-05 | 2.4E-05 | 3.1E-05 | 0.9998 | 0.3321 | 0.7430 | 0.5657 | 0.9334 | 0.9999 |
| SCN3B | NaVβ3 | 1.8E-04 | 1.1E-04 | 3.2E-05 | 4.0E-05 | 0.9998 | 0.0548 | 0.0497 | 0.2831 | 0.3239 | 0.9999 |
| SCN4A | NaV1.4 | 8.4E-05 | 6.3E-05 | 6.4E-05 | 7.0E-05 | 0.9998 | 0.9361 | 0.7969 | 0.7415 | 0.5925 | 0.9999 |
| SCN4B | NaVβ4 | 2.0E-03 | 2.1E-03 | 7.5E-04 | 1.1E-03 | 0.9998 | 0.3261 | 0.7882 | 0.2684 | 0.7315 | 0.9999 |
| SCN5A | NaV1.5 | 3.2E-03 | 3.2E-03 | 1.8E-03 | 2.0E-03 | 0.9998 | 0.8250 | 0.9920 | 0.7406 | 0.8464 | 0.9999 |
| SCN7A | NaV2.1 | 4.3E-04 | 2.9E-04 | 3.0E-04 | 2.4E-04 | 0.9998 | 0.9657 | 0.8509 | 0.5897 | 0.7490 | 0.9999 |
| SCN8A | NaV1.6 | 2.7E-06 | 1.1E-06 | 1.2E-06 | 1.1E-06 | 0.9998 | 0.7315 | 0.6656 | 0.8416 | 0.8018 | 0.9999 |
| SCN9A | NaV1.7 | 5.5E-05 | 2.1E-05 | 8.8E-06 | 1.1E-05 | 0.9998 | NA | 0.0982 | 0.6657 | 0.8642 | 0.9999 |
| SCN10A | NaV1.8 | 1.1E-05 | 8.6E-06 | 3.7E-06 | 3.2E-06 | 0.9998 | 0.5715 | 0.4523 | 0.7601 | 0.6376 | 0.9999 |
| SCN11A | NaV1.9 | 7.9E-07 | 1.9E-06 | 3.4E-07 | 4.9E-07 | 0.9998 | NA | NA | 0.5113 | 0.4996 | NA |

**Cardiac Ion Channels**

**Cardiac Receptors**

| Gene Name | Protein | sSAN | iSAN | RA | LA | padj sSAN vs iSAN | padj sSAN vs RA | padj sSAN vs LA | padj iSAN vs RA | padj iSAN vs LA | padj RA vs LA |
| --- | --- | --- | --- | --- | --- | --- | --- | --- | --- | --- | --- |
| ADRA1A | ADA1A | 2.7E-05 | 1.2E-05 | 2.9E-06 | 4.4E-06 | 0.9998 | 0.0016 | 0.4759 | 0.2197 | 0.7017 | 0.9999 |
| ADRA1B | ADA1A | 3.3E-05 | 3.0E-05 | 2.9E-05 | 1.7E-05 | 0.9998 | 0.7408 | 0.6307 | 0.7656 | 0.7561 | 0.9999 |
| ADRA1D | ADA1D | 1.0E-05 | 1.2E-05 | 4.3E-06 | 9.6E-06 | 0.9998 | NA | 0.7495 | 0.6595 | 0.8980 | NA |
| ADRA2A | ADA2A | 1.5E-03 | 1.2E-03 | 2.6E-04 | 3.0E-04 | 0.9998 | 0.0049 | 0.0947 | 0.0125 | 0.1823 | 0.9999 |
| ADRA2B | ADA2B | 1.3E-03 | 2.2E-03 | 8.5E-04 | 1.6E-03 | 0.9998 | 0.9623 | 0.2455 | 0.5017 | 0.9088 | 0.4839 |
| ADRA2C | ADA2C | 3.5E-03 | 2.9E-03 | 2.1E-03 | 2.9E-03 | 0.9998 | 0.7852 | 0.6097 | 0.9977 | 0.4437 | 0.9999 |
| ADRB1 | ADRB1 | 8.0E-03 | 8.7E-03 | 1.1E-02 | 9.8E-03 | 0.9998 | 0.0649 | 0.0424 | 0.0980 | 0.0619 | 0.9999 |
| ADRB2 | ADRB2 | 2.9E-03 | 2.9E-03 | 1.4E-03 | 1.0E-03 | 0.9998 | 0.2862 | 0.0548 | 0.2281 | 0.0327 | 0.9999 |
| ADRB3 | ADRB3 | 0.0E+00 | 5.2E-05 | 0.0E+00 | 0.0E+00 | 0.9998 | NA | NA | NA | NA | NA |
| AKAP13 | AKP13 | 9.1E-04 | 9.1E-04 | 6.0E-04 | 5.7E-04 | 0.9998 | 0.8763 | 0.9527 | 0.7929 | 0.8671 | 0.9999 |
| ANK1 | ANK1 | 4.6E-04 | 5.3E-04 | 3.8E-04 | 3.5E-04 | 0.9998 | 0.7973 | 0.7351 | 0.9526 | 0.9543 | 0.9999 |
| ANK2 | ANK2 | 4.9E-04 | 5.3E-04 | 3.7E-04 | 3.2E-04 | 0.9998 | 0.8865 | 0.9501 | 0.8980 | 0.8720 | 0.9999 |
| ANK3 | ANK3 | 1.7E-04 | 2.0E-04 | 1.6E-04 | 1.5E-04 | 0.9998 | 0.3139 | 0.3611 | 0.8850 | 0.7890 | 0.9999 |
| ATP2B4 | AT2B4 | 2.2E-03 | 2.1E-03 | 1.2E-03 | 9.9E-04 | 0.9998 | 0.6831 | 0.3693 | 0.7375 | 0.3892 | 0.9999 |
| CAV1 | CAV1 | 1.2E-02 | 1.4E-02 | 1.1E-02 | 1.3E-02 | 0.9998 | 0.6178 | 0.1255 | 0.9403 | 0.3876 | 0.9999 |
| CHGA | CMGA | 3.2E-05 | 2.0E-05 | 2.0E-06 | 1.9E-06 | 0.9998 | NA | NA | NA | NA | NA |
| CHRM1 | ACM1 | 1.3E-05 | 1.2E-05 | 7.9E-06 | 2.9E-06 | 0.9998 | NA | NA | NA | NA | NA |
| CHRM2 | ACM2 | 6.4E-04 | 7.0E-04 | 5.4E-04 | 5.2E-04 | 0.9998 | 0.6599 | 0.5769 | 0.9348 | 0.8541 | 0.9999 |
| CHRM3 | ACM3 | 3.4E-06 | 2.5E-06 | 1.6E-06 | 2.6E-07 | 0.9998 | 0.6660 | 0.0008 | 0.9269 | 0.0136 | 0.3331 |
| CHRNA2 | ACHA2 | 0.0E+00 | 0.0E+00 | 0.0E+00 | 4.3E-07 | NA | NA | NA | NA | NA | NA |
| CHRNA4 | ACHA4 | 4.5E-06 | 9.7E-06 | 3.8E-06 | 2.6E-06 | 0.9998 | NA | NA | 0.7019 | 0.5518 | NA |
| CHRNA7 | ACHA7 | 1.3E-05 | 9.5E-06 | 4.7E-06 | 5.4E-06 | 0.9998 | 0.3436 | 0.6798 | 0.6948 | 0.9225 | 0.9999 |
| CHRNB2 | ACHB2 | 2.9E-05 | 3.8E-05 | 9.5E-06 | 6.6E-06 | 0.9998 | 0.5929 | NA | 0.5319 | 0.3840 | NA |
| CHRNB4 | ACHB4 | 3.8E-06 | 1.4E-06 | 1.0E-06 | 6.5E-07 | 0.9998 | 0.6555 | NA | NA | NA | NA |
| CHRNE | ACHE | 8.4E-04 | 1.1E-03 | 1.1E-03 | 4.6E-04 | 0.9998 | 0.2500 | 0.7718 | 0.6990 | 0.3480 | 0.5906 |
| GNAI2 | GNAI2 | 6.2E-03 | 5.9E-03 | 4.5E-03 | 4.5E-03 | 0.9998 | 0.9274 | 0.5803 | 0.8966 | 0.5218 | 0.9999 |
| MYH6 | MHC-α | 6.4E-01 | 6.8E-01 | 7.7E-01 | 6.6E-01 | 0.9998 | 0.1400 | 0.2358 | 0.2756 | 0.3831 | 0.9999 |
| MYL1 | MLC1 | 6.0E-06 | 2.0E-05 | 1.1E-05 | 1.7E-06 | 0.9998 | 0.5981 | NA | 0.8616 | 0.1580 | NA |
| MYL2 | MLC2 | 4.5E-02 | 5.4E-02 | 7.6E-03 | 2.0E-02 | 0.9998 | 0.3368 | 0.7508 | 0.3706 | 0.6009 | 0.9999 |
| PDE4B | PDE4B | 1.6E-04 | 1.5E-04 | 3.3E-05 | 2.9E-05 | 0.9998 | 0.0295 | 0.0271 | 0.0077 | 0.0135 | 0.9999 |
| PDE4D | PDE4D | 5.5E-05 | 4.7E-05 | 4.4E-05 | 2.9E-05 | 0.9998 | 0.9196 | 0.7102 | 0.7781 | 0.8577 | 0.9999 |
| RAMP3 | RAMP3 | 8.3E-04 | 9.1E-04 | 6.6E-04 | 6.1E-04 | 0.9998 | 0.8643 | 0.8255 | 0.9873 | 0.9557 | 0.9999 |
| RGS2 | RGS2 | 2.6E-02 | 2.4E-02 | 5.8E-03 | 5.3E-03 | 0.9998 | 0.0258 | 0.0565 | 0.0459 | 0.0909 | 0.9999 |
| RGS6 | RGS6 | 3.4E-05 | 4.8E-05 | 3.4E-05 | 2.6E-05 | 0.9998 | 0.6075 | 0.8403 | 0.9448 | 0.7295 | 0.9999 |
| SRC | SRC | 4.1E-04 | 3.3E-04 | 2.4E-04 | 1.8E-04 | 0.9998 | 0.6288 | 0.2400 | 0.9793 | 0.5959 | 0.9999 |
| TNNC1 | TN-C | 4.5E-01 | 5.9E-01 | 5.1E-01 | 5.9E-01 | 0.9998 | 0.2378 | 0.0281 | 0.8150 | 0.2734 | 0.9999 |
| TPM1 | TPM1 | 1.5E-01 | 1.7E-01 | 1.3E-01 | 1.4E-01 | 0.9998 | 0.5572 | 0.0827 | 0.9636 | 0.4920 | 0.9999 |
| VIP | VIP | 1.8E-05 | 3.3E-05 | 8.6E-06 | 1.7E-05 | 0.9998 | NA | NA | NA | 0.9201 | NA |

**Neuronal Proteins**

| Gene Name | Protein | sSAN | iSAN | RA | LA | padj sSAN vs iSAN | padj sSAN vs RA | padj sSAN vs LA | padj iSAN vs RA | padj iSAN vs LA | padj RA vs LA |
| --- | --- | --- | --- | --- | --- | --- | --- | --- | --- | --- | --- |
| CADPS | CAPS1 | 9.0E-05 | 8.6E-05 | 4.7E-05 | 5.4E-05 | 0.9998 | 0.1902 | 0.9145 | 0.3102 | 0.9174 | 0.9999 |
| CHAT | CHAT | 4.1E-06 | 3.3E-07 | 4.9E-07 | 3.6E-07 | 0.9998 | NA | NA | NA | NA | NA |
| CTNNB1 | CTNNB1 | 3.6E-03 | 3.5E-03 | 2.1E-03 | 1.8E-03 | 0.9998 | 0.5510 | 0.2963 | 0.5635 | 0.2732 | 0.9999 |
| DBH | DOPO | 9.1E-06 | 4.2E-06 | 1.4E-05 | 2.4E-06 | 0.9998 | 0.5200 | NA | 0.2960 | NA | NA |
| DDC | DDC | 8.6E-06 | 2.9E-06 | 9.5E-07 | 3.8E-07 | 0.9998 | 0.1001 | 0.0083 | 0.7414 | NA | NA |
| ERBB2 | ERBB2 | 2.5E-03 | 2.8E-03 | 3.2E-03 | 2.8E-03 | 0.9998 | 0.0637 | 0.1979 | 0.2755 | 0.4127 | 0.9999 |
| FZD3 | FZD3 | 9.0E-05 | 7.7E-05 | 8.1E-05 | 5.8E-05 | 0.9998 | 0.5024 | 0.9886 | 0.4295 | 0.7649 | 0.9999 |
| GATA2 | GATA2 | 7.9E-04 | 8.0E-04 | 1.2E-03 | 1.0E-03 | 0.9998 | 0.0665 | 0.0361 | 0.0508 | 0.0179 | 0.9999 |
| GATA3 | GATA3 | 9.0E-05 | 8.2E-05 | 4.2E-05 | 2.9E-05 | 0.9998 | 0.6683 | 0.3835 | 0.6931 | 0.3185 | 0.9999 |
| GBX2 | GBX2 | 1.1E-05 | 0.0E+00 | 4.1E-06 | 0.0E+00 | 0.9998 | NA | NA | NA | NA | NA |
| GDNF | GDNF | 4.5E-05 | 3.8E-05 | 1.5E-05 | 1.0E-05 | 0.9998 | 0.5061 | 0.2277 | 0.6107 | 0.2654 | 0.9999 |
| GFRA2 | GFRA2 | 4.0E-05 | 3.1E-05 | 2.0E-05 | 1.2E-05 | 0.9998 | 0.7737 | 0.3900 | 0.9289 | 0.5244 | 0.9999 |
| GFRA3 | GFRA3 | 9.5E-05 | 5.4E-05 | 3.2E-05 | 5.3E-05 | 0.9998 | 0.4407 | 0.8830 | 0.9368 | 0.6565 | 0.9999 |
| HAND2 | HAND2 | 8.6E-03 | 9.5E-03 | 1.4E-02 | 1.2E-02 | 0.9998 | 0.0000 | 0.0009 | 0.0013 | 0.0158 | 0.9999 |
| NAV2 | NAV2 | 4.7E-05 | 4.2E-05 | 3.0E-05 | 2.3E-05 | 0.9998 | 0.9416 | 0.4807 | 0.9654 | 0.5591 | 0.9999 |
| NEFM | NFM | 7.6E-04 | 4.1E-04 | 1.8E-05 | 2.8E-05 | 0.9998 | 0.0002 | 0.0000 | 0.0106 | 0.0061 | NA |
| NF1 | NF1 | 3.5E-04 | 3.3E-04 | 1.9E-04 | 1.6E-04 | 0.9998 | 0.4868 | 0.2515 | 0.5708 | 0.3148 | 0.9999 |
| NGF | NGF | 1.8E-04 | 1.8E-04 | 1.0E-04 | 7.9E-05 | 0.9998 | 0.7298 | 0.4854 | 0.6451 | 0.3743 | 0.9999 |
| NOS1 | NOS1 | 3.1E-06 | 3.0E-06 | 2.8E-06 | 1.1E-06 | 0.9998 | 0.8042 | 0.5927 | 0.8538 | 0.5753 | 0.9999 |
| NPPA | NPPA | 3.3E+00 | 4.3E+00 | 6.6E+00 | 4.6E+00 | 0.9998 | 0.0039 | 0.0042 | 0.1087 | 0.1803 | 0.9999 |
| NPPB | NPPB | 1.0E+00 | 7.9E-01 | 1.1E+00 | 8.6E-01 | 0.9998 | 0.7967 | 0.8579 | 0.7887 | 0.8199 | 0.9999 |
| NPY | NPY | 8.3E-05 | 2.2E-05 | 1.2E-05 | 7.6E-06 | 0.9998 | 0.3815 | 0.2126 | NA | NA | NA |
| NRP1 | NRP1 | 8.8E-04 | 9.0E-04 | 4.8E-04 | 4.4E-04 | 0.9998 | 0.5399 | 0.5830 | 0.4956 | 0.5137 | 0.9999 |
| NRP2 | NRP2 | 2.9E-04 | 2.3E-04 | 7.1E-05 | 4.8E-05 | 0.9998 | 0.0003 | 0.0157 | 0.0008 | 0.0434 | 0.9999 |
| NTM | NTM | 1.9E-05 | 2.5E-05 | 3.6E-05 | 7.4E-06 | 0.9998 | 0.0901 | 0.3861 | 0.3443 | 0.1151 | 0.0497 |
| NTRK1 | NTRK1 | 5.0E-05 | 4.3E-05 | 1.9E-05 | 2.0E-05 | 0.9998 | 0.2548 | 0.4893 | 0.4121 | 0.6757 | 0.9999 |
| PHOX2A | PHOX2A | 6.2E-05 | 2.5E-05 | 5.5E-06 | 1.7E-06 | 0.9998 | NA | NA | NA | NA | NA |
| PHOX2B | PHOX2B | 1.3E-04 | 3.8E-05 | 0.0E+00 | 4.3E-06 | 0.9998 | 0.0660 | 0.0817 | NA | NA | NA |
| PLXNA4 | PLXNA4 | 9.8E-05 | 9.0E-05 | 5.1E-05 | 2.4E-05 | 0.9998 | 0.4965 | 0.0132 | 0.4359 | 0.0083 | 0.3141 |
| SEMA3A | SEMA3A | 4.4E-06 | 3.1E-06 | 8.5E-06 | 5.5E-06 | 0.9998 | 0.0885 | 0.2673 | 0.1095 | 0.2330 | 0.9999 |
| SEMA3C | SEMA3C | 5.7E-04 | 4.7E-04 | 2.1E-04 | 1.8E-04 | 0.9998 | 0.0887 | 0.0047 | 0.3031 | 0.0947 | 0.9999 |
| SEMA3F | SEMA3F | 5.3E-04 | 5.0E-04 | 3.4E-04 | 3.6E-04 | 0.9998 | 0.8752 | 0.9008 | 0.9260 | 0.8634 | 0.9999 |
| SOX10 | SOX10 | 3.6E-04 | 2.5E-04 | 4.1E-04 | 3.7E-04 | 0.9998 | 0.4943 | 0.3467 | 0.2082 | 0.0810 | 0.9999 |
| SOX11 | SOX11 | 3.7E-04 | 3.1E-04 | 2.6E-04 | 2.3E-04 | 0.9998 | 0.9980 | 0.9748 | 0.8511 | 0.8954 | 0.9999 |
| TBX1 | TBX1 | 3.5E-05 | 2.1E-05 | 2.2E-05 | 1.6E-05 | 0.9998 | 0.9494 | 0.7492 | 0.9007 | 0.9308 | 0.9999 |
| TFAP2A | AP2A | 1.1E-05 | 4.9E-06 | 1.6E-05 | 1.5E-05 | 0.9998 | 0.5247 | 0.3579 | 0.2583 | 0.1020 | 0.9999 |
| TFAP2B | AP2B | 5.6E-07 | 0.0E+00 | 4.9E-07 | 0.0E+00 | NA | NA | NA | NA | NA | NA |
| TH | TH | 1.0E-05 | 3.5E-05 | 3.3E-06 | 4.1E-06 | 0.9998 | NA | NA | NA | NA | NA |
| TP63 | P63 | 1.2E-06 | 1.2E-06 | 2.4E-06 | 1.1E-06 | 0.9998 | 0.2503 | 0.7941 | 0.4795 | 0.8960 | 0.9999 |
| UCHL1 | UCHL1 | 4.5E-03 | 3.2E-03 | 1.0E-03 | 1.1E-03 | 0.9998 | 0.1173 | 0.1596 | 0.2680 | 0.3523 | 0.9999 |
| VCAM1 | VCAM1 | 4.2E-03 | 4.0E-03 | 3.3E-04 | 2.4E-04 | 0.9998 | 0.0159 | 0.0045 | 0.0080 | 0.0023 | 0.9999 |
| VSNL1 | VISL1 | 4.0E-04 | 5.2E-04 | 2.9E-04 | 1.8E-04 | 0.9998 | 0.9792 | 0.4611 | 0.2447 | 0.1104 | 0.9999 |

**Transcription Factors**

| Gene Name | Protein | sSAN | iSAN | RA | LA | padj sSAN vs iSAN | padj sSAN vs RA | padj sSAN vs LA | padj iSAN vs RA | padj iSAN vs LA | padj RA vs LA |
| --- | --- | --- | --- | --- | --- | --- | --- | --- | --- | --- | --- |
| BMP1 | BMP1 | 4.47E-04 | 3.88E-04 | 2.52E-04 | 3.23E-04 | 0.9998 | 0.5575 | 0.7716 | 0.8281 | 0.6410 | 0.9999 |
| BMP2 | BMP2 | 3.85E-03 | 4.03E-03 | 4.18E-04 | 3.42E-04 | 0.9998 | 0.0003 | 0.0000 | 0.0000 | 0.0000 | 0.9999 |
| BMP4 | BMP4 | 1.72E-03 | 1.07E-03 | 5.26E-04 | 6.23E-04 | 0.9998 | 0.0290 | 0.2127 | 0.3063 | 0.8072 | 0.9999 |
| CPNE5 | CPNE5 | 4.64E-04 | 6.53E-04 | 7.27E-04 | 4.41E-04 | 0.9998 | 0.1346 | 0.4173 | 0.5007 | 0.9645 | 0.9999 |
| CSRP2 | CRP2 | 8.86E-04 | 9.14E-04 | 3.34E-04 | 4.39E-04 | 0.9998 | 0.2211 | 0.5449 | 0.2048 | 0.4682 | 0.9999 |
| DLL1 | DLL1 | 7.71E-04 | 9.99E-04 | 7.06E-04 | 5.18E-04 | 0.9998 | 0.5612 | 0.9123 | 0.9490 | 0.4595 | 0.9999 |
| FGFR1 | FGFR1 | 4.03E-03 | 3.48E-03 | 1.95E-03 | 1.89E-03 | 0.9998 | 0.2034 | 0.2216 | 0.4742 | 0.5089 | 0.9999 |
| IGFBP5 | IBP5 | 4.52E-02 | 2.42E-02 | 1.15E-02 | 9.62E-03 | 0.9998 | 0.0025 | 0.0241 | 0.2831 | 0.4055 | 0.9999 |
| ISL1 | ISL1 | 5.68E-05 | 3.51E-05 | 0.00E+00 | 9.71E-07 | 0.9998 | 0.0074 | 0.0080 | NA | NA | NA |
| LBH | LBH | 7.30E-04 | 7.28E-04 | 2.02E-04 | 1.68E-04 | 0.9998 | 0.0004 | 0.0019 | 0.0001 | 0.0013 | 0.9999 |
| NKX2-5 | NKX25 | 3.60E-02 | 4.27E-02 | 4.70E-02 | 4.75E-02 | 0.9998 | 0.0027 | 0.0064 | 0.0941 | 0.0709 | 0.9999 |
| NODAL | NODAL | 2.10E-05 | 7.48E-06 | 1.03E-05 | 8.11E-06 | 0.9998 | 0.8661 | 0.7070 | NA | NA | NA |
| NOTCH3 | NOTC3 | 2.30E-03 | 2.16E-03 | 1.42E-03 | 2.08E-03 | 0.9998 | 0.9196 | 0.4264 | 0.9652 | 0.4258 | 0.9999 |
| PITX2 | PITX2 | 1.99E-04 | 5.56E-05 | 1.98E-04 | 7.43E-04 | 0.9998 | 0.8974 | 0.0017 | NA | 0.0000 | 0.9999 |
| REC114 | REC114 | 1.22E-05 | 1.56E-05 | 2.01E-05 | 5.53E-06 | 0.9998 | 0.2571 | 0.6321 | 0.4901 | 0.3394 | 0.2014 |
| SHOX2 | SHOX2 | 4.48E-03 | 4.27E-03 | 3.99E-04 | 8.36E-04 | 0.9998 | 0.0002 | 0.3613 | 0.0198 | 0.4121 | 0.9999 |
| SLC9A3R2 | NHRF2 | 7.99E-03 | 8.37E-03 | 8.22E-03 | 9.03E-03 | 0.9998 | 0.5727 | 0.2765 | 0.6844 | 0.3570 | 0.9999 |
| SMAD1 | SMAD1 | 1.53E-04 | 1.44E-04 | 9.11E-05 | 8.24E-05 | 0.9998 | 0.6030 | 0.6304 | 0.6568 | 0.6594 | 0.9999 |
| SMAD6 | SMAD6 | 5.55E-04 | 6.93E-04 | 7.83E-04 | 2.69E-04 | 0.9998 | 0.2102 | 0.4985 | 0.5525 | 0.2618 | 0.1729 |
| SMAD9 | SMAD9 | 4.00E-04 | 3.57E-04 | 2.16E-04 | 4.22E-05 | 0.9998 | 0.6933 | 0.0000 | 0.8401 | 0.0000 | 0.0039 |
| SMOC2 | SMOC2 | 3.47E-04 | 3.21E-04 | 1.04E-04 | 1.74E-04 | 0.9998 | 0.0281 | 0.7045 | 0.0419 | 0.7965 | 0.9797 |
| SOX8 | SOX8 | 1.02E-03 | 8.92E-04 | 5.28E-04 | 4.62E-04 | 0.9998 | 0.6366 | 0.6566 | 0.7882 | 0.7831 | 0.9999 |
| TBX3 | TBX3 | 3.01E-03 | 3.03E-03 | 1.20E-03 | 1.17E-03 | 0.9998 | 0.4443 | 0.0203 | 0.9872 | 0.2850 | 0.9999 |
| TBX5 | TBX5 | 2.18E-03 | 2.77E-03 | 2.95E-03 | 2.37E-03 | 0.9998 | 0.1470 | 0.7497 | 0.1132 | 0.9666 | 0.3413 |
| TBX18 | TBX18 | 3.58E-04 | 2.19E-04 | 1.60E-04 | 8.07E-05 | 0.9998 | 0.1622 | 0.0768 | 0.2067 | 0.1144 | 0.9999 |
| TBX20 | TBX20 | 6.72E-04 | 5.79E-04 | 9.28E-04 | 3.71E-04 | 0.9998 | 0.0380 | 0.0596 | 0.3131 | 0.4231 | 0.9999 |
| TENM4 | TN4 | 2.15E-06 | 1.37E-06 | 8.49E-07 | 1.30E-06 | 0.9998 | 0.6696 | 0.9974 | 0.9489 | 0.7338 | 0.9999 |
| VWF | VWF | 1.72E-03 | 2.25E-03 | 1.65E-03 | 1.47E-03 | 0.9998 | 0.5889 | 0.5673 | 0.9691 | 0.9915 | 0.9999 |
| WNT1 | WNT1 | 7.53E-06 | 4.19E-06 | 6.23E-05 | 5.36E-05 | 0.9998 | NA | 0.1373 | 0.1725 | 0.0936 | 0.9999 |
